# Metal‐Organic Framework Based Gas Sensors

**DOI:** 10.1002/advs.202104374

**Published:** 2021-12-22

**Authors:** Hongye Yuan, Nanxi Li, Weidong Fan, Hong Cai, Dan Zhao

**Affiliations:** ^1^ Department of Chemical and Biomolecular Engineering National University of Singapore 4 Engineering Drive 4 Singapore 117585 Singapore; ^2^ State Key Laboratory for Mechanical Behavior of Materials Shaanxi International Research Center for Soft Matter School of Materials Science and Engineering Xi'an Jiaotong University Xi'an 710049 P. R. China; ^3^ Institute of Microelectronics A*STAR (Agency for Science, Technology and Research) 2 Fusionopolis Way, #08‐02 Innovis Tower Singapore 138634 Singapore

**Keywords:** film fabrication, gas sensors, metal‐organic frameworks, molecule sieving, sensing performance, transduction mechanism

## Abstract

The ever‐increasing concerns over indoor/outdoor air quality, industrial gas leakage, food freshness, and medical diagnosis require miniaturized gas sensors with excellent sensitivity, selectivity, stability, low power consumption, cost‐effectiveness, and long lifetime. Metal‐organic frameworks (MOFs), featuring structural diversity, large specific surface area, controllable pore size/geometry, and host‐guest interactions, hold great promises for fabricating various MOF‐based devices for diverse applications including gas sensing. Tremendous progress has been made in the past decade on the fabrication of MOF‐based sensors with elevated sensitivity and selectivity toward various analytes due to their preconcentrating and molecule‐sieving effects. Although several reviews have recently summarized different aspects of this field, a comprehensive review focusing on MOF‐based gas sensors is absent. In this review, the latest advance of MOF‐based gas sensors relying on different transduction mechanisms, for example, chemiresistive, capacitive/impedimetric, field‐effect transistor or Kelvin probe‐based, mass‐sensitive, and optical ones are comprehensively summarized. The latest progress for making large‐area MOF films essential to the mass‐production of relevant gas sensors is also included. The structural and compositional features of MOFs are intentionally correlated with the sensing performance. Challenges and opportunities for the further development and practical applications of MOF‐based gas sensors are also given.

## Graphical abstract

A comprehensive review on the latest progress of metal‐organic framework (MOF)‐based gas sensors relying on different transduction mechanisms is provided. The sensing performance in terms of sensitivity and selectivity is correlated with the structural and compositional features of MOFs and the transduction mechanisms. Critical future directions toward the further development of MOF‐based gas sensors are indicated.

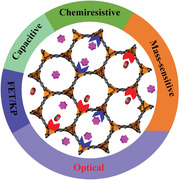

## 1 Introduction

**Figure 1:**
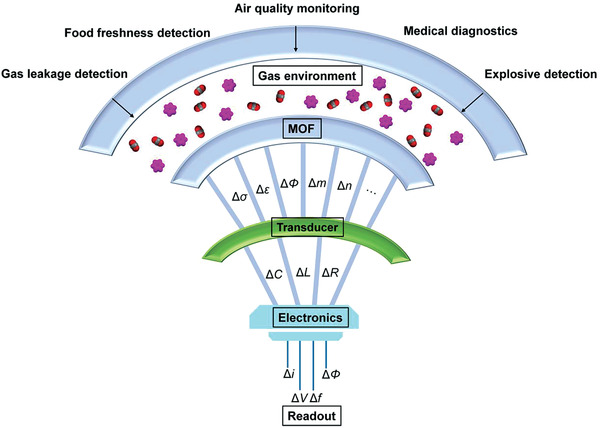
Logic architecture of MOF‐based gas sensors.^[^ ^1c^ ^]^ Gases interact with the organic linkers and/or SBUs of MOFs and thus induce the variation of their physical properties, for example, conductivity (*σ*), permittivity (*ε*), work function (*Φ*), and refractive index (*n*). The transducer converts at least one of the aforementioned quantities into the alteration of electric parameters including capacitance *C*, inductance *L*, and resistance *R*. The circuit with which the sensor couples gives rise to the readout. Signals can be either current (*i*), voltage (*V*), or potential (*E*), and their frequency, magnitude, and/or phase can be measured accordingly. The outmost part summaries the applications of MOF‐based gas sensors. Adapted with permission.^[^ ^1c^ ^]^ Copyright 2019, American Chemical Society.

The perception of physical and chemical information such as touch, pressure, vibration, sight, sound, taste, and smell represents one of the most elegant and formidable mechanisms through which living systems interact with their surroundings.^[^ ^1^ ^]^ Physical organs have developed diverse sensing systems that rely on molecular recognition, thermodynamics, Nernst potential, and photochemical processes to interpret external stimuli into associated signals. Inspired by the functions above, researchers have developed various devices that can probe physical and chemical parameters, for example, motion, pressure, temperature, magnetism, light, color, odor, and even structural fingerprints.^[^ ^1^, ^2^, ^3^ ^]^ Amongst are the gas sensors that have been widely utilized in industrial and household scenarios to detect flammable, toxic, and/or greenhouse gases.^[^ ^4^ ^]^ Gas sensors typically entail two main parts: the sensing material and the transducer. The sensing material interacts with gaseous analytes and afterward induces a fluctuation of its electric, dielectric, magnetic, optical, thermometric, acoustic, colorimetric, and/or gravimetric properties, which can be translated into detectable signals by the transducer (**Figure** **1**). Depending on the transducing mechanism and/or architecture, gas sensors can be mainly subdivided into chemiresistive, capacitive/impedimetric, field‐effect transistor (FET)/Kelvin probe (KP)‐based, electrochemical, gravimetric, and optical sensors.^[^ ^1^, ^5^, ^6^, ^7^, ^8^ ^]^ Remarkable advances on the development of metal oxide semiconductors (MOS),^[^ ^9^ ^]^ carbon‐based materials,^[^ ^10^ ^]^ conducting/semiconducting conjugated polymers,^[^ ^11^ ^]^ MXenes,^[^ ^12^ ^]^ transition metal dichalcogenides,^[^ ^13^ ^]^ piezoelectric ceramics,^[^ ^14^ ^]^ and phosphoranes^[^ ^15^ ^]^ have promoted the fabrication of relevant gas sensors based on such materials. The sensing performance, that is, sensitivity or limit of detection, selectivity, response/recovery time, reproducibility, and stability, is largely governed by the intrinsic nature (e.g., porosity, chemical composition, dimensionality, conductivity, doping, defect, piezoelectricity) of the sensing material, material‐analyte interactions, the adopted fabrication methodologies, and the transducing configuration. The state‐of‐the‐art gas sensors based on the above materials feature apparent advantages and disadvantages.^[^ ^9^, ^10^, ^11^, ^12^, ^13^, ^14^, ^15^ ^]^ Materials with permanent porosity, combined characteristics of inorganic and organic materials, and tunable physicochemical properties are highly sought‐after to fill such gaps.

Metal‐organic frameworks (MOFs), built by inorganic secondary building units (SBUs) and organic ligands via coordination bonds, represent a novel class of crystalline nanoporous materials.^[^ ^16^ ^]^ Various choices of metal nodes and organic linkers endow MOFs with designability in topology, porosity (aperture size and geometry), and even tailor‐made responsiveness to external stimuli, compared to the conventional polymers, zeolites, and other inorganic substances. Therefore, MOFs have been widely exploited for multitudinous applications, including molecular storage/separation,^[^ ^17^ ^]^ catalysis,^[^ ^18^ ^]^ drug delivery,^[^ ^19^ ^]^ and luminescence‐based chemo/bio‐sensing.^[^ ^20^ ^]^ The above unique features also render MOFs suitable candidate materials for gas sensing. Recent progress on the successful implementation of MOFs onto versatile solid‐state surfaces^[^ ^21^ ^]^ propelled the development of MOF‐based devices, for example, electrically‐transduced gas sensors (chemiresistor, capacitors, FETs, mass‐sensitive, and electrochemical sensors), memristors, interferometers, and micro‐ring/surface plasmon resonators.^[^ ^5^, ^6^, ^7^, ^8^, ^22^, ^23^, ^24^, ^25^, ^26^, ^27^, ^28^, ^29^, ^30^, ^31^, ^32^, ^33^, ^34^, ^35^, ^36^, ^37^, ^38^ ^]^ The MOF‐based gas sensors outstand in the following aspects.^[^ ^39^ ^]^ First, the permanent porosity of MOFs offers a large specific surface area and potential active sites (e.g., coordinatively unsaturated open metal sites and terminated functional groups) that are beneficial to elevated gas uptake and transportation. This feature can preconcentrate the targeted analyte and conduce to enhanced sensitivity. Second, the tailor‐made pore size/geometry and physicochemical environment (acidity/alkalinity, hydrophilicity/hydrophobicity, and electron‐rich/electron‐deficient) of MOFs afford selective adsorption toward some specific analytes, and thus lead to higher selectivity and sensitivity. Third, the relatively good crystallinity and predictable periodical arrangement of atoms/molecules within MOFs permit possible structural identification and property correlation associated with the host‐guest interactions from the molecular/atomic level. Fourth, apart from some cases involving strong chemisorption and redox reactions, MOFs’ reversible uptake and release of gases enable the MOF‐based sensors with good regenerability. This is distinctive from the zeolite‐based devices that typically concern a higher energy penalty. Fifth, the relatively high thermal and chemical stability of most MOFs guarantees a long lifetime of MOF‐based sensors. Although recent reviews have discussed either MOF‐based chemical sensors relying on luminescent enhancement/quenching,^[^ ^20^, ^39^, ^40^ ^]^ or MOF‐based sensors only relying on the electrical‐transduction mechanism,^[^ ^1^, ^41^, ^42^, ^43^ ^]^ or open framework materials (including MOFs and covalent organic frameworks) based electronic devices in a broad view,^[^ ^7^ ^]^ a comprehensive review focusing on gas sensing of diverse MOF‐based devices based on the latest literature is absent.

This review summarizes the recent development of MOF‐based gas sensors. We first highlight the latest advances of the integration approaches essential to the preparation of MOF‐based sensors with tangible mass‐production. The device architectures and underlying working principles of MOF‐based gas sensors including chemiresistive, capacitive/impedimetric, FET or KP‐based, mass‐sensitive (e.g., microcantilever, quartz crystal microbalance, microresonator, and surface‐acoustic wave), and optical transduction mechanisms (e.g., Fabry–Pérot interferometers, waveguides or optic Fibers, micro‐ring resonators (MRRs), and surface plasmon resonance/surface‐enhanced Raman scattering (SERS)) are comprehensively outlined. Concurrently, we overview the critical advances on the sensing application of MOF‐based sensors from both analytical and historical perspectives. The pertinent roles of MOFs within such sensors are discussed, and their structural and compositional features are correlated with the sensing performance. We conclude by providing insights on the challenges and opportunities facing the further development and practical applications of MOF‐based gas sensors.

## 2 Integration Methods of MOFs onto Solid‐State Surfaces in Large‐Area

The past two decades have witnessed great success in designing, synthesizing, and characterizing novel MOFs in powder form with intriguing physicochemical properties. However, the key lies in the shaping of MOFs onto solid‐state substrates to expand their applications in advanced technologies, including electronic device‐based chemical sensing and membrane‐based separation. Diverse methods have been reported so far on the preparation of MOF films onto various surfaces, including direct hydro/solvothermal growth, direct deposition assisted by spin/dip‐coating,^[^ ^44^ ^]^ layer‐by‐layer or stepwise growth,^[^ ^45^ ^]^ electrochemical deposition,^[^ ^46^ ^]^ interfacial synthesis approach coupled with a subsequent transfer,^[^ ^32^, ^47^ ^]^ and template‐mediated growth/transformation.^[^ ^48^ ^]^ Films with variable morphologies, thickness, adhesive property, and/or preferential orientation can be obtained relying on the fabrication method adopted and process variables. This field has been well‐documented so far, and several recent reviews have summarized different aspects of this topic, including film orientation and patterning.^[^ ^21^, ^49^, ^50^, ^51^ ^]^ Our objective within this section is to highlight the latest progress (studies published after 2019) on the large‐area fabrication of MOF films, which is absent from the current reviews but crucial to the practical industrial applications of MOF‐based gas sensors.

Fabrication of MOF films in large‐area necessitates the wide usage of MOFs for industrial scenarios. However, forming large‐area (typically at least a few cm^2^) MOF films with good homogeneity, tunable thickness, appreciable mechanical property, and preferential orientation remains arduous. This is because the formation of the majority of MOFs relies on the hydro/solvothermal approach, and the area of MOF films obtained by the direct growth/deposition method is largely limited by the volume of the vessel commonly utilized. In the meanwhile, the poor processability of most MOF powders also restricts the fabrication of large‐area MOF films.^[^ ^49^, ^50^, ^51^ ^]^ We hereafter summarize several approaches that permit the fabrication of large‐area MOF films. Their respective features are available in **Table** **1**.

**Table 1:** Summary of different approaches suitable for large‐area fabrication of MOF films

Fabrication method	Suitable substrate	Operation	Film feature (size, thickness, orientation)	Large‐area fabrication	Ref
Spin‐coating	Flat surfaces	Synthesis of MOF particles, deposition by spin‐coater at RT	Controllable and size‐dependent thickness, typically non‐orientated	Yes, depending on the spin‐coater	^[^ ^44^ ^]^
Electrochemical	Conductive surfaces	In situ formation of MOF particles and electrochemical deposition on anode/cathode	Controllable thickness (thin) by varying current density, time, etc., non‐orientated	Yes, vessel‐dependent	^[^ ^50^, ^52^, ^53^, ^54^ ^]^
Interfacial synthesis	Flat surfaces	First synthesis of 2D film in combination with transfer technique	Controllable thickness by varying the precursor concentration, time, additive, etc., Orientated	Yes, vessel‐dependent	^[^ ^55^ ^]^
Template‐mediated growth/transformation	Depending on the shape of the template	First deposition of template and then conversion	Controllable thickness, non‐orientated	Yes, template‐dependent	^[^ ^48^, ^56^, ^57^, ^58^, ^59^ ^]^
Doctor‐blading or spray‐coating	Flat surfaces	Synthesis of solution processable MOF suspension, deposition by doctor‐blading or spay‐coating	Controllable thickness, orientation depending on the dimensionality of MOFs	Yes, depending on the doctor‐blader or spray‐coater	^[^ ^60^, ^61^ ^]^

### 2.1 Spin‐Coating Approach

One straightforward way to fabricate large‐area MOF films involves the direct deposition of preformed nanometered MOF particles onto flat surfaces by spin‐coating.^[^ ^44^ ^]^ The method first requires the synthesis of MOF particles and then the dispersion of MOF particles in some volatile solvents. A solution layer containing MOF particles can be spin‐coated onto the surface, and solvent evaporation affords a uniform and polycrystalline MOF layer. The thickness of MOF films largely depends on the concentration and viscosity of the MOF solution, as well as the crystal size of MOFs and process variables (e.g., spin speed and time). This approach has been widely utilized in wafer‐scale microfabrication of functional oxide and photoresist polymer layers. Nevertheless, the key drawbacks associated with this approach are the lack of regular arrangement of MOF crystals and the presence of boundaries. Whereas the latter is detrimental to the charge/mass transport and device performance.

### 2.2 Electrochemical Approach

**Figure 2:**
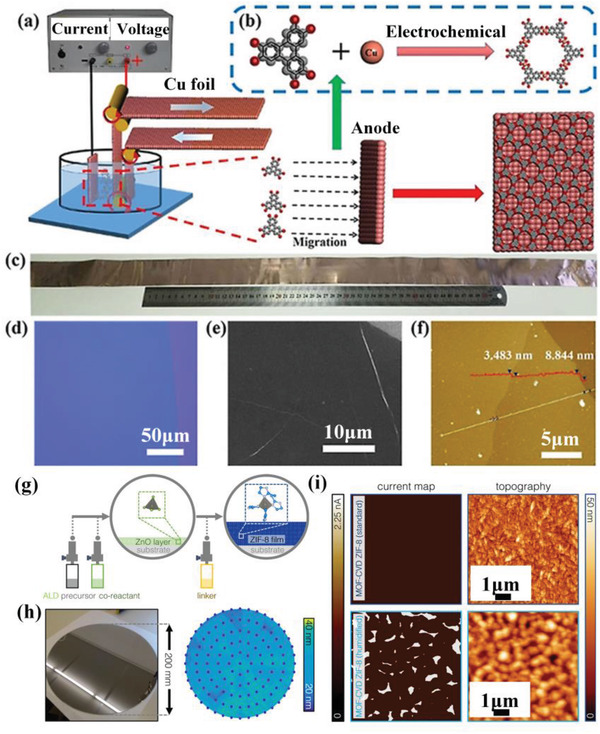
a) Electrolytic cell utilized for the fabrication of Cu_3_(HHTP)_2_ films on Cu (100) foils. b) Schematic representation of the coordination between Cu^2+^ ions and the deprotonated organic linkers. c) Digital photograph of a Cu_3_(HHTP)_2_ film‐coated Cu foil. d) Optical image of the Cu_3_(HHTP)_2_ film. e) Representative scanning electron microscopy (SEM) image of the Cu_3_(HHTP)_2_ film. f) Atomic force microscopy (AFM) image of the Cu_3_(HHTP)_2_ film. Reproduced with permission.^[^ ^54^ ^]^ Copyright 2020, Wiley‐VCH. g) Schematic representation of the MOF‐CVD process. h) Digital photograph of the ZIF‐8 thin film fabricated on an 8‐inch wafer and the corresponding ellipsometry mapping in thickness. i) Current and topography maps captured by conductive AFM of the resultant ZIF‐8 films deposited under standard (top) and humid conditions (bottom). The locations where current is detectable represent pinholes within the ZIF‐8 layer. Reproduced with permission.^[^ ^59^ ^]^ Copyright 2019, American Chemical Society.

Alternatively, large‐area MOF films can also be harvested by the electrochemical method. Since the seminal work conducted by researchers from BASF,^[^ ^52^ ^]^ great success has been made toward preparing MOF films on either the anodic or the cathodic surfaces where a bias voltage is applied, by modulating the solution composition and/or the type of plates.^[^ ^21^, ^50^ ^]^ Isolated MOF crystals and/or compact layers composed of intergrown MOF crystallites (e.g., HKUST‐1, ZIF‐8, MIL‐100, MIL‐53) can be obtained facilely by tuning the solution composition, voltage, current density, and/or deposition time.^[^ ^50^, ^53^ ^]^ Recently, Liu et al. demonstrated the fabrication of large‐area Cu_3_(2,3,6,7,10,11‐hexahydroxytriphenylene (HHTP))_2_ MOF films on single crystal Cu (100) anodes via a controllable electrochemical assembly approach (**Figure** **2**a,b).^[^ ^54^ ^]^ The atomically flat Cu anode surface facilitates the controllable release of Cu^2+^ ions under a suitable voltage. The Cu^2+^ ions coordinate with the pre‐deprotonated organic linkers, resulting in homogeneous and large‐area Cu_3_(HHTP)_2_ films with tunable thickness (Figure 2c–f) on the anode surfaces just via varying the voltage. The resultant Cu_3_(HHTP)_2_ film exhibits a substantially enhanced electrical conductivity compared with the film prepared by the interfacial synthesis approach (see Section 2.3 for detailed information). Note that conductivity was calculated according to the law of resistance (*R* = *ρ L*∙*S* ^−1^, where *ρ* stands for the resistivity and is reciprocal to conductivity, *L* denotes the channel length, and *S* denotes the sectional area. The *R* value was obtained by the typical I‐V curves). Notably, the deposition will terminate once the MOF film covers the whole anodic/cathodic surface, and this method is only amenable to conductive substrates. This issue somehow precludes its practical application in MOF‐based electronic devices that rely on complicated configurations, and needs to be well addressed in future studies.

### 2.3 Interfacial Synthesis Approach

The interfacial synthesis approach coupled with subsequent transfer techniques, for example, Langmuir–Blodgett trough, also permits the formation of large‐area MOF films onto various flat surfaces.^[^ ^55^ ^]^ Within the realm of interfacial synthesis, coordination between metal centers and organic ligands occurs at the air/liquid or liquid/liquid interfaces. In contrast, for the latter, the reactant is dissolved separately in two immiscible solvents. Confined formation of layered structures at the interface allows for their further lateral growth of 2D frameworks over a large length scale, and the liquid “substrate” promotes the spontaneous arrangement of the building blocks. The subsequent transfer of the atomically‐flat nanosheets with uniform morphology and larger lateral size onto solid‐state surfaces under increased surface pressures by Langmuir–Blodgett trough leads to the fabrication of ultimate nanodevices with unique functionalities and potential mass‐production.

### 2.4 Template‐Mediated Growth/Transformation Approach

Additionally, the template‐mediated growth/transformation has also been introduced by several different groups to prepare MOF films in large‐area on various substrates, including flexible fibers,^[^ ^56^ ^]^ copper hydroxide nanobelts,^[^ ^48b^ ^]^ nickel foams,^[^ ^57^ ^]^ and silicon wafers.^[^ ^58^, ^59^ ^]^ This approach typically entails two steps. The first step concerns the direct preparation of intermediate layers such as metals, inorganic salts, metal oxides, and metal hydroxides as the template to provide the metal sources for constructing the targeted MOFs. The second step typically relates to the controllable dissolution of the preformed layers under a suitable hydro/solvothermal condition and further coordination with the organic ligands, resulting in the formation of MOF films. Comparatively, as indicated in Figure 2g, the so‐called MOF‐CVD (chemical vapor deposition) process pioneered by Ameloot et al. undergoes a vapor‐solid transformation of the deposited metal oxide to yield the desired MOF with a noticeable volume expansion.^[^ ^59^ ^]^ Various types of MOF films, for example, ZIF‐8, ZIF‐7, ZIF‐67, ZIF‐72 (ZIFs stands for zeolitic imidazolate frameworks), Al(OH)(1,4‐naphthalenedicarboxylate (NDC)), Cu(1,4‐benzodicarboxylate (BDC)), Ni‐NDC, MOF‐74, HKUST‐1, MOF‐5, UiO‐66, and CuCDC, have been successfully fabricated onto variable substrates.^[^ ^48^, ^49^, ^58^, ^59^ ^]^ As exemplified in Figure 2h,i, the wafer‐scale fabrication of thin and pinhole‐free ZIF‐8 films with great surface homogeneity and thickness down to dozens of nanometers can be achieved in a non‐wet chemistry fashion. The solvent‐free MOF‐CVD approach circumvents the potential chemical contamination, and is compatible with the widely‐used photolithography during microfabrication. Thus, it holds great promise for integrating MOFs onto patterned microelectronic devices with feasible mass‐production and cost‐effectiveness.

### 2.5 Doctor‐Blading or Spray‐Coating

**Figure 3:**
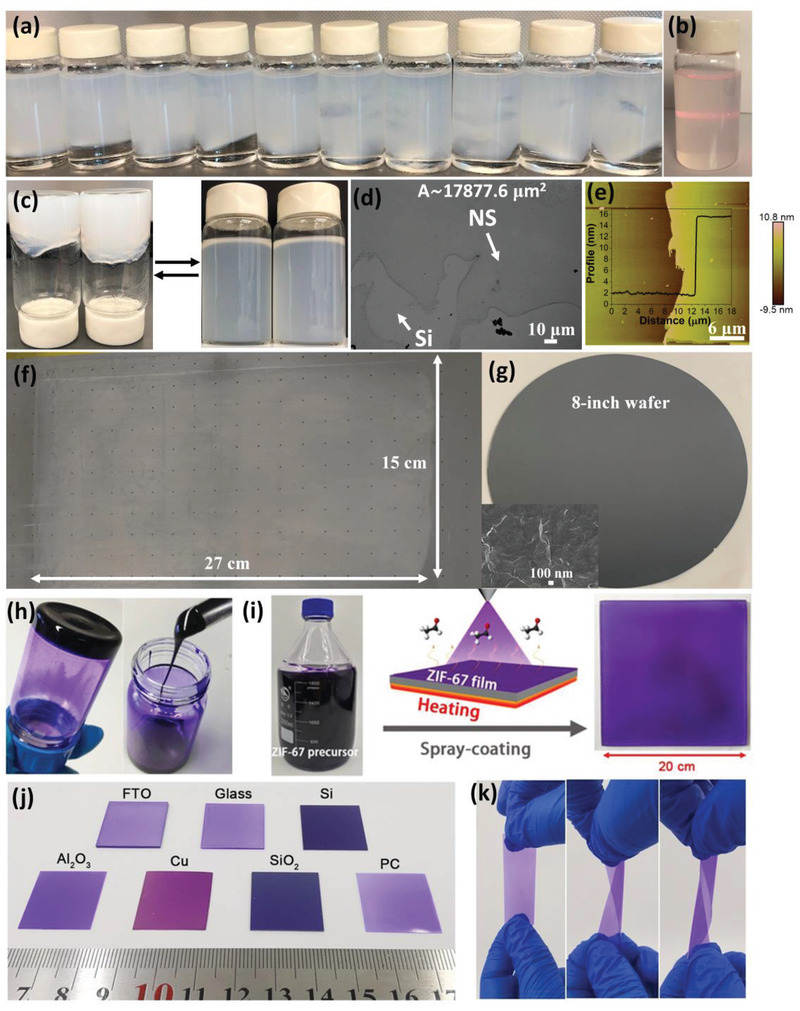
a,b) Digital photographs of the as‐synthesized NUS‐8 suspensions consisting of large NUS‐8 nanosheets. c) The reversible transformation from NUS‐8 sol to gel upon removing or adding solvent. d) Representative SEM image of the monodispersed NUS‐8 nanosheet on Si surface. e) Representative AFM image of monodispersed NUS‐8 nanosheet with its corresponding height profile. f) Digital photograph of NUS‐8 film fabricated on PS substrate. g) Digital photograph of NUS‐8 thin film fabricated on 8‐inch wafer. Reproduced with permission.^[^ ^60^ ^]^ Copyright 2021, Wiley‐VCH. h) Digital photograph of the ZIF‐67 sol. i) Spray‐coating process for fabricating large‐area ZIF‐67 film. j) Digital photographs of the fabricated ZIF‐67 films on different substrates. k) Digital photographs of ZIF‐67 films on PC substrate under variable twisting. Reproduced with permission.^[^ ^61^ ^]^ Copyright 2021, Wiley‐VCH.

Recently, demonstrations on the fabrication of large‐area MOF films have also been reported by utilizing the solution processability of MOF suspensions. We directly synthesized highly stable NUS‐8 suspensions with variable functional terminated groups by adding capping molecules and tuning the concentrations of precursors and MOF‐solvent interactions (**Figure** **3**a–c).^[^ ^60^ ^]^ The resultant NUS‐8 nanosheets with an averaged area of above 15 000 µm^2^ and a thickness of around 13 nm (Figure 3d,e) gelated through weakly and non‐covalently bonded networks and exhibited excellent solution processability. As such, large‐area and textured NUS‐8 films (Figure 3f,g) with tunable thickness and appreciable mechanical property can be facilely fabricated by just drop‐casting or doctor‐blading. Simultaneously, Yu et al. reported the direct synthesis of MOF colloidal solutions composed of ultra‐small amorphous nanoparticles (Figure 3h,i). Polycrystalline ZIF‐67 films with tunable thickness can be facilely prepared in large areas onto various substrates by a spray‐coating approach (Figure 3i,j).^[^ ^61^ ^]^ This method also took the best advantage of the excellent solution processability of amorphous MOF suspensions, and induced the transformation of amorphous MOF nanoparticles to ZIF‐67 crystallites under low‐temperature in situ heating. Interestingly, the ZIF‐67 coated polycarbonate (PC) displays good flexibility and robustness under rigorous twisting (Figure 3k) without damaging the MOF film on top. The above studies provide novel perspectives for simply fabricating large‐area MOF films. More efforts need to be spared in developing generic approaches for synthesizing MOF suspensions possessing excellent solution processability.

## 3 MOF‐Based Devices for Gas Sensing

The working principles of MOF‐based gas sensors depend primarily on molecular recognition and the subsequent transduction of the recognition events into detectable signals.^[^ ^1^ ^]^ The former involves the interaction between the targeted analyte and MOFs that feature large specific surface areas, controllable pore sizes/geometries, and host‐guest interactions. Distinctive from the non‐ordered networks (e.g., polymers), MOFs with precisely tuned pore environments offer unique advantages in selective molecular recognition ascribed to the well‐known molecular sieving effect. The latter relates to the fluctuation induced by the above interactions in electric, dielectric, magnetic, optical, thermometric, acoustic, colorimetric, and gravimetric properties, which can be interpreted into measurable signals by the transducer. Noteworthily, the interactions are typically non‐covalent interactions, including van der Waals forces, hydrogen bonding, and *π*‐*π* interactions, which enable reversible uptake and release toward the analyte and thus reversible sensing events.^[^ ^39^ ^]^ However, possible covalent bonding and/or redox reaction between the targeted analyte and the chosen reactive MOF may induce irreversible response yet higher selectivity and sensitivity.^[^ ^31^ ^]^ This review only focuses on MOF‐based devices for gas sensing, which can be classified into several types of sensors based on transducing mechanisms, as discussed in the following. Representative MOF‐based devices relying on different working principles along with their sensing characteristics are summarized in **Table** **2**.

**Table 2:** Representative MOF‐coated devices for gas sensing

Working principle	Active material	Targeted gas, detection range [ppm]	Sensing condition	LOD [ppm]	Ref
Chemiresistive	ZIF‐8/ZIF‐7 coated ZnO	Acetone 0.25–100	260 °C Variable humidity	0.0019	^[^ ^26^ ^]^
Chemiresistive	ZIF‐8 coated ZnO	Formaldehyde 100	300 °C Variable humidity	NA	^[^ ^63b^ ^]^
Chemiresistive	ZIF‐67 Co(IM)_2_	Formaldehyde 5–500 Trimethylamine 2–50	150 °C 75 °C	NA NA	^[^ ^67^, ^78^ ^]^
Chemiresistive	Cu_3_(HITP)_2_	NH_3_ 0.5–10	RT	NA	^[^ ^23^ ^]^
Chemiresistive	Ni_3_(HITP)_2_	VOCs 200	RT	NA	^[^ ^22^ ^]^
Chemiresistive	NiPc‐MOF NiNPc‐MOF	NH_3_ 2–80 H_2_S 0.2–80 NO 0.02–1	RT Variable humidity	NH_3_ 0.31–0.33 H_2_S 0.02–0.033 NO 0.001–0.0011	^[^ ^31^ ^]^
Chemiresistive	Cu‐TCPP on Cu‐HHTP	NH_3_ 100 Benzene 1–100	RT	NA	^[^ ^79^ ^]^
Capacitive	Al‐BDC, Fe‐BTC, Cu‐BTC, Li‐doped Fe‐BTC, and Fe‐doped Fe‐BTC	Humidity 0–2.5%	120–240 °C 1 Hz	NA	^[^ ^86^ ^]^
Capacitive	MIL‐125‐NH_2_	Humidity 11–95%	RT, 100 Hz	NA	^[^ ^87^ ^]^
Capacitive	RE‐fcu‐MOF	H_2_S 0.1–100	RT	0.005	^[^ ^30^ ^]^
Capacitive	Mg‐MOF‐74	CO_2_ 200–5000 Benzene 2–100	RT, 500 kHz	NA	^[^ ^27^ ^]^
Capacitive	Cu_3_(BTC)_2_	VOCs 0–1000	RT, 1 MHz	7.3 (Methanol) 150.5 (Ethanol)	^[^ ^89^ ^]^
FET/KP	Cu_3_(HHTP)_2_	NH_3_ 500	RT	NA	^[^ ^80^ ^]^
FET	Diketopyrrolopyrrole/Cd(NDC)_0.5_(PCA) MOF	Explosive compounds	RT	NA	^[^ ^101^ ^]^
FET	PDVT‐10/[Ni(TPyP)‐(TiF_6_)]_n_	NO_2_ 0.025–50	RT	0.0083	^[^ ^102^ ^]^
KP	HKUST‐1	Aldehydes 10–50	RT	NA	^[^ ^97^ ^]^
FET/KP	UiO‐66‐NH_2_	DMMP 0.003–0.015 0.04–0.15	RT	0.0003	^[^ ^96^ ^]^
Microcantilever	HKUST‐1	Humidity 0.18–0.98% CO_2_ 0–70%	RT	NA	^[^ ^34^ ^]^
Microcantilever	MOF‐5, HKUST‐1, ZIF‐8, and MOF‐177	*p*‐xylene 0.4–250	RT	0.4 (HKUST‐1)	^[^ ^106^ ^]^
Microcantilever	MIL‐53	CO_2_ 1–100%	RT	NA	^[^ ^111^ ^]^
QCM	Cu_2_(ndc)_2_(dabco)	VOCs NA	RT	NA	^[^ ^118^ ^]^
QCM	HKUST‐1	Humidity 5–75%	RT	NA	^[^ ^117a^ ^]^
QCM	ZIF‐8	5.3–26.5%	RT	NA	^[^ ^120^ ^]^
Microresonator	ZIF‐69	CO_2_ 0–9000	RT	15	^[^ ^123^ ^]^
SAM	HKUST‐1	Humidity 3–14 800 ppmv	RT	NA	^[^ ^127^ ^]^
SAM	MFU‐4 MFU‐4l	CO_2_ 10–100%	RT	NA	^[^ ^126^ ^]^
SAM	ZIF‐67 ZIF‐8	Acetone 5–25 Ethanol 5–25 NH_3_ 5–25	RT	NA	^[^ ^128^ ^]^
Fabry–Pérot	ZIF‐8	Propane 0–100% Ethanol in water 0–100%	RT	0.3% (Ethanol in water)	^[^ ^37^ ^]^
Fabry–Pérot	HKUST‐1/silica composite	Humidity 0–12 000 Ethanol 0–12 000 CS_2_ 0–12 000	RT	NA	^[^ ^129^ ^]^
Fabry–Pérot	UiO‐66	VOC 0–100%	RT	NA	^[^ ^133^ ^]^
Optic Fiber	HKUST‐1	Humidity 2.5–10 ppmv	RT	NA	^[^ ^135^ ^]^
Optic Fiber	ZIF‐8	CO_2_ 10–100%	RT	NA	^[^ ^136^ ^]^
MRR	ZIF‐8	VOC NA	RT Variable humidity	Styrene 0.099 Toluene 0.076 Benzene 0.035 Propylene 0.029 0.058 (Methanol)	^[^ ^38^ ^]^
SPR	HKUST‐1/Ag	CO_2_ 0–100%	RT	NA	^[^ ^144^ ^]^
SPR	ZIF‐8 ZIF‐93	VOCs	RT	2.5 (Methanol) 73 (*n*‐BuOH)	^[^ ^145^ ^]^
SERS	ZIF‐8 coated FON	Benzene 0–100%	RT	540	^[^ ^147^ ^]^
SERS	ZIF‐8/Ag	CO_2_ 0.05–500	RT	ppb‐level	^[^ ^148^ ^]^
SERS	MIL‐100	VOCs	RT	2.5 (Benzene)	^[^ ^150^ ^]^

Note that RT stands for room temperature.

### 3.1 MOF‐Based Chemiresistive Sensing

**Figure 4:**
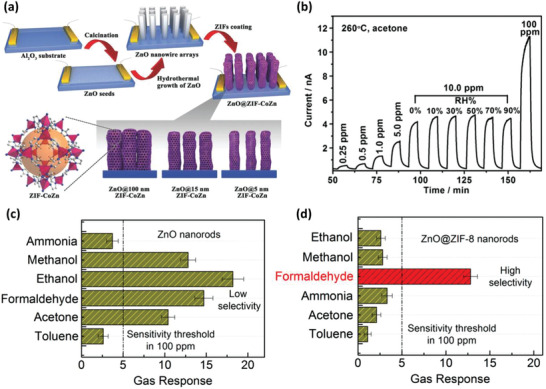
a) Schematic representation of ZIF‐8 coated ZnO gas sensors. b) Dynamic response/recovery curve of the ZIF‐8 coated ZnO gas sensor upon exposure to variable concentrations of acetone at the presence of variable humidity levels. The thickness of the ZIF‐8 layer is 5 nm. Reproduced with permission.^[^ ^26^ ^]^ Copyright 2016, Wiley‐VCH. Sensing selectivity of ZnO‐based sensors c) without and d) with ZIF‐8 coating under exposure to 100 ppm of different analytes at a working temperature of 300 °C. Reproduced with permission.^[^ ^63b^ ^]^ Copyright 2015, American Chemical Society.

The working principle of MOF‐based chemiresistive sensors relies on the analyte‐induced alteration in electrical conductance of the sensing material upon uptake of the targeted analyte.^[^ ^22^, ^23^, ^24^, ^25^ ^]^ The interaction between the sensing material and the targeted analyte alters the conductance of the sensing material through modulating the concentration and/or mobility of charge carriers (electrons or holes) of MOFs under exposure to variable concentrations of gas. Depending on the physicochemical nature of the gaseous analyte and/or type, relevant correlations between the variation in conductance of the sensing material and gas concentration can be therefore made. However, unlike the conventional MOS,^[^ ^9^, ^62^ ^]^ semiconductive organic polymers,^[^ ^11^ ^]^ and layered carbon‐based materials,^[^ ^10^ ^]^ most MOFs intrinsically exhibit low electrical conductivity due to the limited concentration of charge carriers and relatively poor mobility.^[^ ^1b^ ^]^ Given that, thin MOF layers with narrowed aperture size were intuitively coated outside the active layers of conventional MOS gas sensors as filter layers to permit selective passthrough of some gases.^[^ ^26^, ^63^ ^]^ The working principle of this scenario leans on the redox reaction between the chemisorbed oxygen species on MOS and the targeted reducing or oxidizing gases at the gas‐solid interface under appropriate heating. The MOF shell layer serves as the filter layer to sieve and preconcentrate the targeted analyte, therefore overcoming the inherent drawbacks (e.g., low selectivity) of the MOS‐based gas sensors to some extent. Based on this strategy, several different groups have reported in parallel the fabrication of heterostructured MOF@MOS as chemiresistive gas sensors.^[^ ^26^, ^63^, ^64^ ^]^ For example, Xu et al. presented the fabrication of hydrophobic ZIF‐8/ZIF‐67 coated zinc oxide (ZnO) sheath‐core nanowires through a wet chemistry approach.^[^ ^26^ ^]^ A seeded ZnO layer was first prepared and then grew into nanowired ZnO, partial of which served as the template for the formation of hydrophobic ZIF‐8/ZIF‐67 film (**Figure** **4**a). The gas‐sensing performance of ZIF‐8/ZIF‐67 shelled ZnO‐based sensors toward variable concentrations of acetone vapor was investigated under the presence of moisture. The dynamic response/recovery curve demonstrated improved selectivity of the sheath‐core nanostructure toward acetone, compared with the bare ZnO nanowire‐based sensors (Figure 4b). More intriguingly, the presence of moisture exerted no impact on the sensing performance attributed to the hydrophobic nature of the MOF structure.

Parallel studies conducted by Kim et al.^[^ ^63a^ ^]^ and Fan et al.^[^ ^63b^ ^]^ also indicated that ZIF‐8 coated ZnO‐based sensors showed substantially enhanced selectivity, thanks to the molecular sieving effect of the ZIF‐8 framework with a narrow aperture size of 3.4 Å. As depicted in Figure 4c,d, the ZIF‐8 coated ZnO sensors exhibited decreased sensitivity toward various gases (100 ppm) at 300 °C. Still, the selectivity toward formaldehyde over other gases is higher than the pristine nanoroded ZnO‐based sensors.^[^ ^63b^ ^]^ Gases with kinetic diameters far exceeding 3.4 Å can hardly enter the pores of ZIF‐8. In consequence, large molecules, for example, toluene, can scarcely be detected, as demonstrated in Figure 4d. Those studies justify the pertinent role of MOFs in sensing applications with improved performance via molecular sieving effect. However, the sensors based on heterostructures are typically confronted with high‐power consumption limited by the transduction mechanism of MOS‐based sensing.

**Figure 5:**
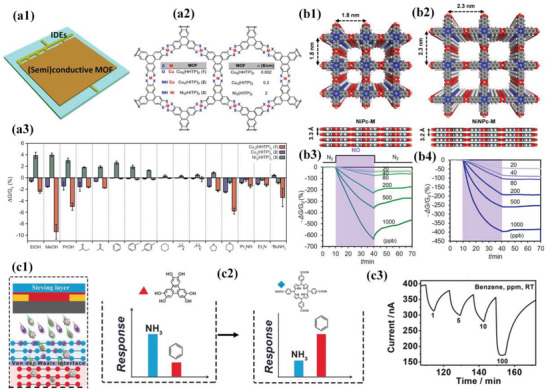
a1) Representative architecture of (semi)conductive MOF‐coated chemiresistive gas sensors. Note that the MOF films can be fabricated via drop‐casting, in situ growth, spray‐coating, etc. a2) Representative (semi)conductive MOFs constructed by Cu^2+^ or Ni^2+^ with HITP or HHTP used for gas sensing. a3) Sensing responses of MOF‐based sensor array toward different types of VOCs at 200 ppm after 30 s exposure. Δ*G* denotes the variation in conductance, whereas the *G*o stands for the original conductance. Reproduced with permission.^[^ ^22^ ^]^ Copyright 2015, American Chemical Society. b1,b2) Schematic representation of isoreticular MOFs built respectively by phthalocyanine (Pc) and naphthalocyanine (NPc) ligands. Dynamic responses of Ni‐Pc‐MOF b3) and Ni‐NPc‐MOF based sensors b4) toward different concentrations of NO at RT. Reproduced with permission.^[^ ^31^ ^]^ Copyright 2018, American Chemical Society. c1) Schematic representation of Van der Waals heterostructured MOF‐on‐MOF based gas sensing. c2) Tunable selectivity of the heterostructured MOF‐on‐MOF based gas sensor toward benzene and NH_3_. c3) Dynamic response of the MOF‐on‐MOF based gas sensor toward variable concentrations of benzene at room temperature. Reproduced with permission.^[^ ^79^ ^]^ Copyright 2019, Wiley‐VCH.

Apart from compositing with other conductive molecules,^[^ ^65^ ^]^ polymers and/or carbon‐based materials for chemiresistor sensing,^[^ ^66^ ^]^ MOFs featuring (semi)conductive nature can also be directly utilized for chemiresistive sensing (**Figure** **5**a 1). By monitoring the conductivity of such MOF layers deposited onto electric circuits (typically composed of IDEs) under exposure to different types of analytes, one can realize such a sensing function. Various types of (semi)conductive MOFs, such as ZIF‐67,^[^ ^67^ ^]^ TUB‐75,^[^ ^68^ ^]^ M_3_(2,3,6,7,10,11‐hexaiminotriphenylene (HITP) or HHTP)_2_,^[^ ^22^, ^23^, ^24^, ^25^, ^69^ ^]^ Cu_3_(hexaiminobenzene (HIB))_2_,^[^ ^70^ ^]^ MBHT,^[^ ^71^ ^]^ M2,3,9,10,16,17,23,24‐octamethoxyphthalocyaninato M(II) (NiPc)‐M or M3,4,12,13,21,22,30,31‐octahydroxynaphthalocyaninato M(II) (NiNPc)‐M (M stands for Ni, Co, Cu or Fe),^[^ ^31^ ^]^ and other quasi‐graphene MOFs with extended *π*‐conjugated networks have been reported so far,^[^ ^72^, ^73^, ^74^ ^]^ showing somehow (semi)conductivity. Achieving (semi)conductivity in MOFs requires maximizing the density and/or mobility of charge carriers.^[^ ^75^ ^]^ The generation of charge carriers within MOFs can be realized via photo or thermal excitation, exotic doping, and electron/hole injection. The charge mobility/transport relies on three primary ways, that is, hopping, through‐bond, and through‐space.^[^ ^75^ ^]^ Several reviews have been reported so far, summarizing different design principles for building MOFs and/or MOF‐based composites with certain electrical conductivity.^[^ ^75^, ^76^, ^77^ ^]^ One major progress is designing and synthesizing planar 2D conjugated MOFs with graphene‐resemble architectures that show considerable (semi)conductivity.^[^ ^75^ ^]^ These reported structures are generally built by octahedral or square planar transition metal ions (Ni, Cu, Co, or Fe) with conjugated (benzene, triphenylene, phthalocyanine (Pc)‐based) organic linkers with redox‐active ortho‐substituted hetero atoms (N, O, S). Metal nodes with unpaired electrons and organic linkers with radical moieties can act as the delocalized charge sources and permit the potential charge transport within the 2D plane attributed to the enhanced conjugation and *p*‐*d* orbital coupling. The electronic structure and thus conductivity can be tuned via the choices of charge state and identity of the metallic nodes, as well as the symmetry and functional group of organic ligands. Their conductivity varies from 10^−9^ to 2.5 × 10^3^ S cm^−1^ at RT, depending on the MOF type, crystal quality, and the testing method/configuration.^[^ ^1b^ ^]^ Availability of these materials enables the direct fabrication of portable (semi)conductive MOF‐based chemiresistive gas sensors with cost‐effectiveness, low power consumption, and pertinent sensing performance.

Zhang et al. first reported the usages of semiconductive ZIF‐67 and Co(imidazole (IM))_2_ for MOF‐based chemiresistive sensing toward variable concentrations of formaldehyde and trimethylamine at 150 and 75 °C, respectively.^[^ ^67^, ^78^ ^]^ The ZIF‐67 based sensor showed selective and linear detection toward formaldehyde in the range of 5–500 ppm over other gaseous analytes, such as methanol, humidity, methane, and ammonia. On the other hand, the Co(IM)_2_ based sensor exhibited selective detection toward trimethylamine in the range of 2–50 ppm, probably attributed to the appreciable interaction between the targeted analyte and Co(IM)_2_. Meanwhile, several research groups have reported the usages of so‐called (semi)conductive MOFs for chemiresistive sensors that can operate at RT. Dincă et al. presented the first Cu_3_(HITP)_2_‐based chemiresistive sensors fabricated by drop‐casting the Cu_3_(HITP)_2_ dispersion onto relevant substrates.^[^ ^23^ ^]^ The resultant sensors exhibited linear and reversible detection toward sub‐ppm levels of ammonia vapor at room temperature. Conversely, the Ni_3_(HITP)_2_‐based sensor showed negligible response toward ammonia under identical conditions, highlighting the tunability of gas sensing performance of the MOF‐based sensors via the rational design of MOFs. Their following work demonstrated the sensor array fabrication based on several conductive triphenylene‐based MOFs that share an identical topology (Figure 5a, 2).^[^ ^22^ ^]^ The sensor array, fabricated either by drop‐casting or solvent‐free mechanical drawing, exhibited disparate discrimination toward variable types of volatile organic compounds (VOCs) at 200 ppm (Figure 5a, 3). The authors claimed that the charge transfer and the hydrogen bonding between the 2D MOF and the tested analytes accounted for the observed distinguishable sensing behaviors. Other researchers, including Xu,^[^ ^24^, ^79^ ^]^ Mirica,^[^ ^1^, ^25^, ^31^ ^]^ Martí‐Gastaldo,^[^ ^80^ ^]^ Kitagawa,^[^ ^81^ ^]^ and Deng,^[^ ^82^ ^]^ also fabricated a series of (semi)conductive MOFs‐based chemiresistive sensors relying on rigid and/or flexible substrates, which significantly promoted the development of this field.

Recently, Mirica et al. designed and synthesized isoreticular nickel Pc‐ and nickel naphthalocyanine (NPc)‐linked conductive 2D MOFs, named NiPc‐MOF and NiNPc‐MOF (Figure 5b, 1, 2), respectively.^[^ ^31^ ^]^ The above MOFs were successfully drop‐casted onto interdigitated gold (Au) electrodes for chemiresistive sensing toward variable concentrations of NO (Figure 5b, 3, 4), H_2_S, and NH_3_. As depicted in Figure 5b, 3, 4, the NiPc‐MOF and NiNPc‐MOF coated gas sensors were all able to detect variable concentrations of NO in the ppb range within tens of minutes. Simultaneously, the sensitivity of NiPc‐MOF coated sensor toward NO seems much higher compared with that of the NiNPc‐MOF coated one. This MOF‐based sensor showed exceptionally low detection limits at room temperature of around 1.05, 26.5, and 320 ppb for NO, H_2_S, and NH_3_, respectively. Additionally, the presence of moisture (5000 ppm) hardly changed the sensing performance. Such a performance surpasses those of other sensing materials, including MOS and metal dichalcogenides.^[^ ^1b,c^ ^]^ The observed sensing performance was correlated with the charge transfer interactions induced by the targeted gases captured within MOFs, which can be verified by X‐ray photoelectron spectroscopy and electron paramagnetic resonance spectroscopy. This work provides novel perspectives for designing and synthesizing (semi)conductive MOFs targeted for chemiresistive sensing. However, issues such as cross‐sensitivity between different analytes, scale‐up production, and long‐term stability of relevant sensors need to be well addressed before their practical application in complicated sensing scenarios.

To address the cross‐sensitivity of (semi)conductive MOF‐based sensors toward different analytes, Xu et al. reported the fabrication of heterostructured MOF‐on‐MOF‐based chemiresistive sensors via van der Waals interaction induced stacking (Figure 5c).^[^ ^79^ ^]^ Specifically, a (semi)conductive Cu‐HHTP layer as the sensing layer was first prepared by layer‐by‐layer spraying onto Au interdigitated electrodes functionalized with hydroxyl‐terminated groups. Multiple‐layered Cu‐5,10,15,20‐tetrakis(4‐carboxyphenyl)porphyrin (TCPP) nanosheets synthesized by a one‐pot approach were afterward transferred on top of the preformed Cu‐HHTP layer via stamping. Heterostructured MOF‐on‐MOF thin film was formed due to the van der Waals interaction. The Cu‐HHTP based sensor was able to detect both NH_3_ and benzene vapor, with a higher sensitivity toward NH_3_ than benzene vapor (Figure 5c, 2). The insulating Cu‐TCPP layer with abundant coordinatively‐unsaturated open Cu sites interacted strongly with NH_3_ molecules and thus restrained their further diffusion to the underneath Cu‐HHTP layer. Therefore, the Cu‐TCPP on Cu‐HHTP based chemiresistive sensor showed substantially enhanced selectivity at room temperature toward benzene over NH_3_ than that of the bare Cu‐HHTP based sensor (Figure 5c, 3). This study demonstrates the synergetic impacts of heterostructured MOF architectures with cascading properties targeted for chemiresistive sensing.

### 3.2 MOF‐Based Capacitive/Impedimetric Gas Sensing

The working principle of such sensors relies on alternating current (AC) electrical circuits with capacitors composed of two conductive electrodes separated by a dielectric or insulating layer.^[^ ^5^, ^7^, ^27^ ^]^ The impedance *Z* in the AC mode is defined in analogy to DC resistance, that is, the ratio of voltage to current (Equation (1)).^[^ ^7^ ^]^ The impedance *Z* can be divided into two parts: the resistance *R* as the real part, and the capacitive reactance *X*_C_ as the imaginary part. Thereinto, the capacitive reactance (*X*_C_) is oppositely proportional to capacitance *C* (Equation (2)), which can be expressed as a function of the permittivity (*ɛ*_r_) of the sensing material, the vacuum permittivity (*ɛ*_o_), the electrode area, and the distance *d* between the separated electrodes (Equation (3)). Note that Equation (3) represents the basic definition of capacitor composed of two vertically stacked conductive electrodes separated by an insulating layer or air. The capacitance of the sensor can thus be derived in Equation (4), whereas *ƞ* stands for the number of the IDE fingers; *l* and *t* represent the length and thickness of the electrodes, respectively.^[^ ^83^ ^]^

(1) $$Z\;=\;\frac{V(t)}{I(t)}\;=\;\mathrm{ReZ}\;+\;\mathrm{ImZ}\;=\;R\;+\;\mathrm{jXC}$$

(2) $$\mathrm{XC}\;=\;-\frac{1}{2\pi \mathrm{fC}}$$

(3) $$C=\varepsilon_{r}\frac{\varepsilon_{o}A}{d}$$

(4) $$C_{\mathrm{sensor}}=\eta \varepsilon_{r}\frac{lt}{d}$$

**Figure 6:**
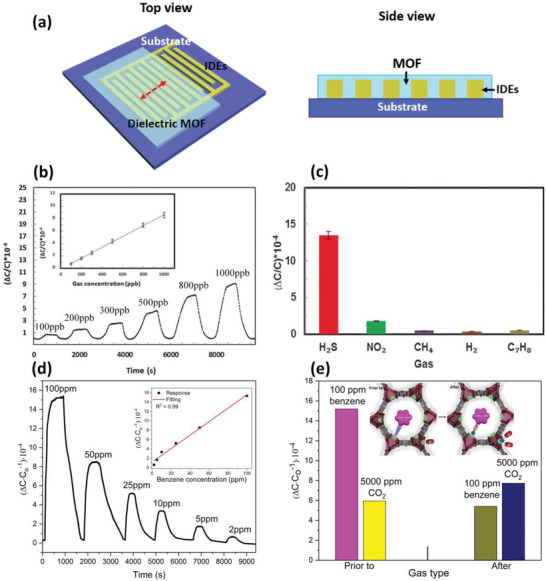
a) Representative schematic views of the dielectric MOF‐based capacitive device. Reproduced with permission.^[^ ^60^ ^]^ Copyright 2021, Wiley‐VCH. b) Dynamic response of RE‐fcu‐MOF based sensor toward variable concentrations of H_2_S. c) Selectivity of the fcu‐MOF based sensor toward different analytes. The concentration of the tested gases was 10 ppm. Reproduced with permission.^[^ ^30^ ^]^ Copyright 2016, Wiley‐VCH. d) Dynamic response of Mg‐MOF‐74 based capacitive sensor toward different concentrations of benzene at room temperature. e) Saturated responses of the Mg‐MOF‐74 based capacitive sensor toward benzene and CO_2_ before and after ethylenediamine modification. Reproduced with permission.^[^ ^27^ ^]^ Copyright 2019, Wiley‐VCH.

The majority of MOFs show an intrinsically insulating nature due to the limited concentration of charge carriers and/or mobility, which greatly limits their usages in chemiresistive gas sensing. Instead, insulating MOFs can be deposited onto the area between parallelly separated interdigitated electrodes to serve as the dielectric layer (**Figure** **6**a).^[^ ^60^ ^]^ Accordingly, the permittivity or dielectric constant or impedance of the MOF layer varies under uptake or interaction with various gaseous analytes in variable concentrations due to the change in local polarity. In such a fashion, the fluctuation of capacitance/impedance can be correlated with the measured gas concentration.^[^ ^27^, ^28^, ^29^ ^]^ Obviously, from the device's perspective, the distance *d* between the separated electrodes plays an essential role in regulating the capacitance. For instance, it is feasible to make a minor variation of permittivity detectable by decreasing the *d* distance so that sensitivity can be increased accordingly.^[^ ^84^ ^]^ IDEs with nanogaps may not apply to the above equations. Still, they can make the best use of the highest electric fields adjacent to the protruding parts/edges of IDEs, thus contributing mostly to the measured capacitance system. From the material point of view, MOFs with tunable pore size, geometry, and functionality can preconcentrate and sieve the targeted analyte. Deservedly, the dielectric MOF‐based capacitive sensors outperform the conventional ceramic‐ and polymer‐based capacitive sensors that typically exhibit low sensitivity and selectivity.^[^ ^85^ ^]^

Moos et al. reported one of the earliest examples of MOF‐based chemicapacitors.^[^ ^86^ ^]^ Several commercially available MOF powders from BASF, including Al‐BDC, Fe‐benzene‐1,3,5‐tricarboxylic acid (BTC), Cu‐BTC, Li‐doped Fe‐BTC, and Fe‐doped Fe‐BTC, were individually cast together with organic binders onto IDEs and were tested separately for humidity sensing.^[^ ^86^ ^]^ The Fe‐BTC‐based capacitive/impedimetric sensor registered the best sensing performance and featured an approximately linear sensitivity of 590 MΩ vol%^−1^ (normalized impedimetric sensor signal by the humidity level) toward humidity in the range of 0–2.5 vol% at 1 Hz and 120 °C. Similarly, Ruan et al. described the usage of NH_2_‐functionalized MIL‐125(Ti) for impedimetric sensing toward variable levels of moisture at room temperature.^[^ ^87^ ^]^ The NH_2_‐MIL‐125(Ti) coated humidity sensor showed a modest response toward 11 to 95% RH at 100 Hz with excellent linearity and relatively fast response/recovery (< 50 s). Another example reported by Qiu et al. presented the usage of a continuous thin Cu_3_(BTC)_2_ film with a thickness of 1 µm for capacitive gas sensing.^[^ ^88^ ^]^ The Cu_3_(BTC)_2_ film was grown onto the polished Cu plate, and aluminum (Al) was afterward deposited as the circled electrode. The resultant Cu_3_(BTC)_2_‐based sensor exhibited a fast (< 60 s) and linear response of around 1.499 pF per % RH toward humidity (between 11.3 and 84.3%) at 1 MHz. Intriguingly, the sensor also showed a fast and full recovery within 20 s. The authors attributed the fast response and recovery of the Cu_3_(BTC)_2_‐based sensor to the fast kinetics of the physisorption process of the Cu_3_(BTC)_2_ film toward moisture. Analogous studies reported by Zeinali et al. also concerned the Cu_3_(BTC)_2_‐based capacitors targeted to detect VOCs, including methanol and ethanol.^[^ ^89^ ^]^ The capacitors based on Cu_3_(BTC)_2_ (with a thickness of ≈ 5 µm) showed linear responses at room temperature and 1 MHz toward methanol and ethanol, both in the range of 0–1000 ppm. The detection limits for methanol and ethanol were derived to be 47.3 and 150.5 ppm, respectively, which are approximately four orders of magnitude lower than that of the earlier MOF‐based devices. At the same time, the response/recovery usually took several minutes, distinctive from that of the earlier studies (only dozes of seconds).^[^ ^88^ ^]^ Utilizing the same MOF, De Smet et al. presented the fabrication of capacitors via in situ electrochemical deposition of Cu_3_(BTC)_2_ films with a thickness of around 5–7 µm on substrates composed of Cu IDEs.^[^ ^90^ ^]^ The resultant capacitive sensors showed fast (≈ 150–300 s) and reversible responses toward methanol and humidity in the range of 100–8000 ppm at 30 °C and 20 kHz. The dynamic response followed diffusion‐mediated kinetics, whereas the saturated response matched well with the Langmuir adsorption model. Simultaneously, a higher sensitivity toward humidity than methanol was observed, probably due to its stronger affinity to the MOF framework. Fabrications of pure CAU‐10^[^ ^91^ ^]^ and NH_2_‐MIL‐53(Al)/polymer^[^ ^92^ ^]^ composite‐based capacitors have also been reported, showing good potential in gas monitoring.

This field has also been greatly advanced by other researchers.^[^ ^27^, ^28^, ^29^, ^30^, ^60^, ^93^, ^94^ ^]^ Eddaoudi, Salama, et al. reported the direct solvothermal growth of rare‐earth (RE)‐based MOF thin films featuring fcu topology (RE‐fcu‐MOF) on modified Au IDEs targeted for H_2_S sensing. The capacitor‐based on oriented RE‐fcu‐MOF film exhibited a sensitive detection at room temperature toward variable concentrations of H_2_S (Figure 6b),^[^ ^30^ ^]^ with a detection limit of 5 ppb extrapolated from the quasi‐linear response‐gas concentration curve. The sensor also showed a highly selective detection toward H_2_S over other analytes, including NO_2_, CH_4_, H_2_, and C_7_H_8_ (Figure 6c).

Later, the same team reported the fabrications of a series of capacitive sensors based on NDC‐Y‐fcu‐MOF,^[^ ^93^ ^]^ MFM‐300,^[^ ^94^ ^]^ or MIL‐96 films,^[^ ^95^ ^]^ respectively targeted for the detections of NH_3_, SO_2_, methanol, or humidity. The above sensors exhibited outstanding sensing performance to some extent in terms of sensitivity and selectivity at RT, with detection limits ranging from ppm to ppb levels. Sooner, Zhao et al. presented the fabrication of capacitive sensors based on Mg‐MOF‐74 with coordinatively unsaturated metal open sites.^[^ ^27^ ^]^ The in situ solvothermal growth of Mg‐MOF‐74 films onto substrates with IDEs was achieved by judicious choice of metal to ligand ratio. The Mg‐MOF‐74 based capacitive sensors after activation demonstrated a quasi‐linear detection toward benzene vapor in the range of 2–100 ppm (Figure 6d), as well as toward CO_2_ (200–5000 ppm) at room temperature. Post‐synthesis functionalization upon the Mg‐MOF‐74 films with ethylenediamine substantially decreased the sensitivity toward benzene vapor by around 60%, whereas increased sensitivity toward CO_2_ by around 25% (Figure 6e). The observed declined sensitivity toward benzene was ascribed to reduced porosity and *π*‐complexation‐driven Lewis acid‐base interactions between the benzene molecules and the *π*‐cloud entities. In contrast, the tailor‐made CO_2_‐amine interaction was responsible for the increased sensitivity toward CO_2_ due to the enhanced adsorption capacity. The study demonstrates the tunability of sensing performance of MOF‐based sensors via subtle control of host (MOF)‐guest (targeted gases) interactions. The following work reported by the same group describes the on‐chip conversion of metal hydroxide layers to Ni‐NDC MOF films utilized for capacitive gas sensing.^[^ ^29^ ^]^ The capacitors based on the converted Ni‐NDC MOF with appreciable adhesion property exhibited modest sensitivity toward benzene vapor at RT. Meanwhile, the sensors showed good selectivity toward benzene over other gases, including CO_2_, CH_4_, and C_3_H_8_, attributed to their disparate permittivities and differentiated gas‐MOF interactions. Recently, Zhao et al. realized the direct synthesis of solution‐processable NUS‐8 nanosheets functionalized with different terminated groups (‐NH_2_, ‐CH_3_, ‐H) and fabricated a series of relevant capacitors accordingly.^[^ ^60^ ^]^ It was demonstrated that the capacitors integrated with NUS‐8 thin films with different functional groups showed distinctive sensing behaviors toward acetone in the ppm range. Specifically, the amine‐functionalized NUS‐8 coated sensors exhibited a higher sensitivity toward acetone than the rest. The disparate sensing performance was assigned to distinguishing host‐guest interactions verified by molecular dynamics simulations. More importantly, this study demonstrates the advantage of solution‐processable MOFs on fabricating electronic devices, paving the way for the potential mass‐production of relevant sensors.

### 3.3 MOF‐Based Field‐Effect Transistors and/or Kelvin Probes for Gas Sensing

**Figure 7:**
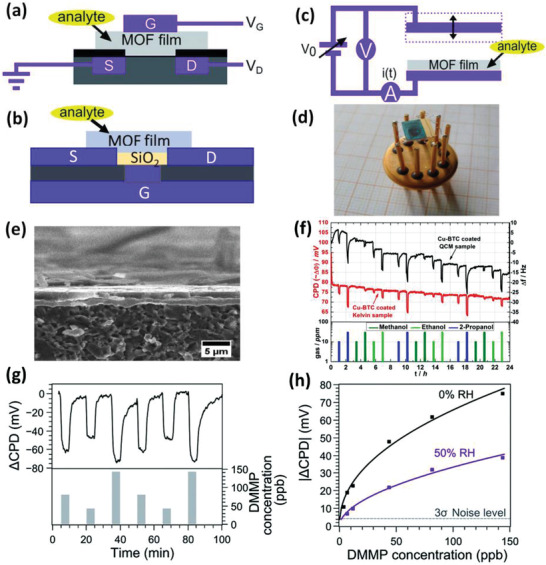
Schematic representations of FET sensors based on a) dielectric MOFs and b) (semi)conductive MOFs. c) Schematic representation of KP sensors based on MOFs. a,c) Reproduced with permission.^[^ ^96^ ^]^ Copyright 2016, Royal Society of Chemistry. d) Digital photograph of Cu‐BTC‐based KP sensor. e) Cross‐sectional SEM image of the Cu‐BTC film. f) Real‐time response of the Cu‐BTC coated KP sensor toward variable analytes, in comparison with that of the Cu‐BTC coated QCM sensor. Reproduced with permission.^[^ ^100^ ^]^ Copyright 2014, American Chemical Society. g) Real‐time CPD response of the UiO‐66‐NH_2_ coated KP sensor toward DMMP in the range of 40–150 ppb. h) Freundlich isotherm fittings of the CPD response respectively at 0% and 50% RH. Reproduced with permission.^[^ ^96^ ^]^ Copyright 2016, Royal Society of Chemistry.

FETs are composed of three conducting electrodes (i.e., source, drain, and gate), a semiconductor, and a dielectric layer, by which the gate is separated from the drain and the source (**Figure** **7**a,b).^[^ ^96^ ^]^ The flow of current (*I*_DS_) between the source and the drain terminals can be modulated by applying a voltage to the gate (*V*_GS_). The drain current (*I*_DS_), as the readout in the FET devices, leans largely on the gate‐source voltage (*V*_GS_), drain‐source direct‐current voltage *V*_DS_, mobility of charge carrier in the drain‐source channel (*µ*), the width to length ratio of the above channel (*W L* ^−1^), the normalized capacitance of the insulator (*C*), and the threshold voltage (*V*_T_). While, the *V*_T_ is correlated with the difference between the sensing channel and the work functions of the gate. It can be derived from the *I*_DS_‐*V*_GS_ transfer characteristics and is also dependent on the analyte concentration.^[^ ^7^ ^]^

(5) $$I_{\mathrm{DS}}=\mu C\frac{W}{L}[\left(V_{\mathrm{GS}}-V_{T}\right)V_{\mathrm{DS}}-\frac{V_{\mathrm{DS}}^{2}}{2}]$$

The underlying working mechanism of FET sensors depends on the modulation of the current flow of channel materials and/or gate electrode work function via the variation in surface modification and/or adsorbed species. Variations of work function of (semi)conductive materials can be feasibly probed using the KP method.^[^ ^32^ ^]^ Within the KP configuration, a plate capacitor is built composing of an analytical electrode and a vibrating reference electrode (Figure 7c).^[^ ^96^ ^]^ The KP measures the contact potential difference (CPD) between the stationary electrode and the reference electrode. The CPD stems from a bulk contribution related to the Fermi level of the two electrodes and a surface contribution associated with the adsorption of molecules. As such, the real‐time variation of the work function can be monitored to reflect the concentration of the analyte. A few studies have described the fabrication of FET devices based on (semi)conductive MOFs with conjugated frameworks, with a focus on detecting gaseous analytes.^[^ ^80^, ^96^, ^97^, ^98^, ^99^, ^100^ ^]^ For instance, Martí‐Gastaldo et al. reported the fabrication of Cu_3_(HHTP)_2_‐based FET devices and studied the origin of chemiresistive sensing toward NH_3_.^[^ ^80^ ^]^ The interaction between NH_3_ and the Cu sites led to the distortion of the framework and thus the variation of the bandgap was considered as the underlying origin for the chemiresistive response. This was verified by experimental results and computational modeling. Studies on the fabrication of FET sensors based on composites of organic semiconductors and insulating MOFs were also reported by different groups. Rao et al. described the combination of semiconducting diketopyrrolopyrrole with Cd(NDC)_0.5_(4‐pyridinecaboxylic acid (PCA)) MOF as FET sensors targeted to detect nitro‐based explosive compounds.^[^ ^101^ ^]^ The composite‐based FET sensors showed sensitivities at RT of 1.7 and 27.5 µA for ppb‐level of 1,3,5‐trinitro‐1,3,5‐triazacyclohexane and 2,4,6‐trinitrotoluene, respectively. Eddaoudi, Salama, and co‐workers reported the combination of organic semiconductors with thiophene donor blocks (named PDVT‐10) with an n‐type fluorinated 3D MOF with a formula of [Ni(5,10,15,20‐tetra(4‐pyridyl)porphyrin (TPyP))‐(TiF_6_)]_n_ utilized for FET‐based sensing toward trace NO_2_.^[^ ^102^ ^]^ The chosen MOF acted as the preconcentrating layer toward NO_2_; and the organic polymer with electron donors served as the channel layer, through which electrons can be potentially transferred or interacted with the electron‐deficient molecules to induce the change in conductivity. Impressively, the relevant sensors showed stable and substantially increased sensitivity (by six times) toward NO_2_, compared with that of the pristine organic polymer. The detection limit of the sensor was derived to be 8.25 ppb, and the sensor also exhibited a negligible response toward a wide range of humidity (5–90%).

The usage of dielectric MOFs coated on (semi)conducting electrodes for FET or KP‐based sensing has also been reported (Figure 7a,c ). Davydovskaya et al. described the coating of Cu‐BTC MOF on TiN electrodes. They demonstrated its CPD responses toward trace aldehydes, including hexanal, ethanal, pentanal, and propanal, under the presence of other interfering gases.^[^ ^97^ ^]^ The Cu‐BTC coated sensors exhibited selective detection toward ppm‐level pentanal at ambient condition, with a response time of 3–11 min depending on the tested concentration. The presence of oxygen exerted a negligible impact on the sensing performance, whereas the presence of moisture above 30% RH dramatically affected the work function response toward pentanal. Minor responses toward ethanal, propanal, and hexanal than pentanal were observed, ascribed to their varied polarity and size.^[^ ^97^ ^]^ In a follow‐up study, they evaluated the sorption behaviors of Cu‐BTC films toward ppm‐levels of various alcohol vapors such as 1‐propanol, 2‐propanol, methanol, and ethanol, both via quartz crystal microbalance (QCM) and work‐function based KP (Figure 7d–f).^[^ ^100^ ^]^ The Cu‐BTC coated QCM and KP sensors with an averaged MOF thickness of around 2 µm all showed comparable responses toward the above alcohols at ppm‐level (Figure 7f). The sensing results demonstrated that the CPD response increased as the length of the alcohol chain increased, which can also be verified by mass‐sensitive QCM sensors. Additionally, the presence of water molecules greatly affected the interaction between the analyte and the unsaturated metal nodes of the Cu‐BTC framework, and thus modified the selectivity of relevant sensors. Complementary studies on the fabrications of KP sensors coated with a series of MOFs, including M‐MOF‐74 (M stands for Mg, Zn, Co, or Ni) and Zn‐BTC, have also been reported from the same group.^[^ ^99^, ^103^ ^]^

Using the same approach, Ameloot et al. recently described the fabrication of UiO‐66‐NH_2_ coated FET sensors to detect ppb‐level nerve agent simulant dimethyl methylphosphonate (DMMP).^[^ ^96^ ^]^ This work demonstrated a sensitive and reversible response toward ppb‐level DMMP (Figure 7g), with a detection limit of around 0.3 ppb extrapolated from the response‐concentration curve. Such a value is among the lowest ever reported in the literature. Impressively, the UiO‐66‐NH_2_ coated sensor was able to detect 2 ppb DMMP even under 50% RH (Figure 7h). Density functional theory (DFT) calculation on the interaction pair between DMMP and the MOF framework suggested that the interaction between DMMP and the amino groups of organic ligands, as well as the interaction between DMMP and the Zr‐OH/Zr‐OH_2_ clusters accounted for the noticeable response toward DMMP. This work justified the judicious choice of MOFs for selective binding/adsorption of the targeted analyte that can substantially increase the sensitivity and selectivity of relevant sensors. Although the KP method allows for the fabrication of relevant sensors based on the vast majority of dielectric MOFs, the major issue lies in the difficulty of miniaturizing oscillating electrodes, making it challenging for large‐scale applications of KP sensors.^[^ ^5^ ^]^

### 3.4 MOF‐Based Mass‐Sensitive Gas Sensing

**Figure 8:**
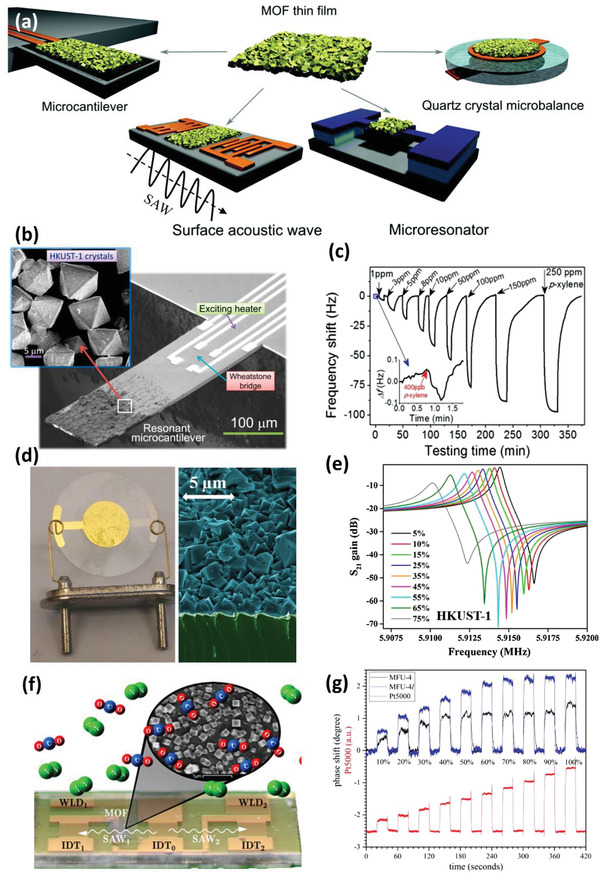
a) Schematic overview of the four types of MOF‐based mass‐sensitive gas sensors discussed. Reproduced with permission.^[^ ^5^ ^]^ Copyright 2017, Royal Society of Chemistry. b) SEM image of HKUST‐1 coated microcantilever sensor. The inset depicts the zoomed‐in surface morphology of the MOF coating. c) Dynamic response curve of the HKUST‐1 coated microcantilever sensor toward variable concentrations of *p*‐xylene. The inset displays the response curve toward 400 ppb *p*‐xylene. Reproduced with permission.^[^ ^106^ ^]^ Copyright 2016, American Chemical Society. d) Digital photograph of the HKUST‐1 coated QCM sensor. The right column presents the cross‐sectional SEM image of the HKUST‐1 film. e) Representative frequency spectrum of the HKUST‐1 coated QCM sensor toward variable moisture concentrations. Reproduced with permission.^[^ ^117a^ ^]^ Copyright 2017, Elsevier. f) Schematic representation of MFU‐4 coated SAW sensor. The inset presents the SEM image of the as‐grown MOF film, composed of isolated crystals. g) Measured phase shift of the MOF‐coated SAW sensors toward variable concentrations of CO_2_, compared to that of a resistive thermal conductivity sensor. Reproduced with permission.^[^ ^126^ ^]^ Copyright 2017, American Chemical Society.

Mass‐sensitive gas sensing relies on the transduction of gaseous molecules captured within the active layer into mechanical responses, for example, variation in vibration behavior or bending, which can be translated into readable signals, typically via piezoelectric approaches. Different devices have been reported so far for gas sensing through monitoring the variation of mass of the active layer, including microcantilever, QCM, microresonator, and surface acoustic wave (SAW) (**Figure** **8**a).^[^ ^5^ ^]^ Compared with the conventional polymer‐based mass‐sensitive gas sensors, the MOF‐based sensors provide more superior sensing performance in terms of sensitivity and selectivity, which can be attributed to the attractive features of MOFs. The following parts describe the working principles of such MOF‐based mass‐sensitive gas sensors and highlight some examples reported so far.

#### 3.4.1 MOF‐Based Microcantilevers for Gas Sensing

Microcantilever‐based gas sensors measure either the deflection of the microcantilever in static mode or the variation in resonant frequency of the oscillating microcantilever in dynamic mode, both of which are induced by the uptake of gaseous molecules within the active layer coated on the microcantilever.^[^ ^5^, ^34^ ^]^ The uptake of gaseous molecules induces stress localized at the interface between the active layer and the beam surface, making the cantilever bend. Such bending can be quantitatively monitored by position‐sensitive detectors, diode lasers, piezoresistive strain gauges, or capacitive sensing. The deflection can be quantitatively expressed according to Stoney's equation.^[^ ^104^ ^]^

(6) $$\Delta z=\frac{3L^{2}\;\left(1-\nu \right)}{{\mathrm{Et}}^{2}}\;\delta \sigma$$

Where *L* and *t* represent the length and thickness of the microcantilever, respectively. *E* and *ν* denote Young's modulus and Poisson ratio, respectively. *δσ* stands for the differential surface stress induced by the molecule captured. Whereas in the dynamic mode, the sensor response related to the mass variation can be expressed as below:^[^ ^104^ ^]^

(7) $$\Delta f\;=\;\frac{f_{n}}{2m_{e}}\Delta m$$

Where Δ*f* denotes the variation in frequency induced by the molecule adsorbed (Δ*m*); *f*_n_ and *m*_e_ represent the resonant frequency and effective mass of the resonator, respectively. Scaling down the dimension of the resonator can substantially increase its sensitivity. Combining highly porous materials like MOFs with the microcantilevers results in more significant changes in mass or resonant frequency upon guest uptake. Selective adsorption of specific analytes toward MOFs with adjustable porosity (aperture size and geometry) and functionality can also improve selectivity. Such a concept was primarily adopted by Allendorf, Hesketh, and co‐workers to detect moisture via the combination of HKUST‐1 films with static microcantilevers equipped with piezoresistive sensors.^[^ ^34^ ^]^ Prompt (within seconds) and selective detection toward H_2_O or CO_2_ can be reached via modulating the hydration state of the axial Cu(II) sites within the MOF framework. It was also indicated that the expansion of crystal lattice was responsible for stress‐induced chemical detection. A following comprehensive theoretical study conducted by Allendorf, Hesketh, and co‐workers described detailed discussions on the influence of mechanical properties of MOFs (e.g., density, Poisson's ratio, and Young's Modulus) on the gas sensing performance of microcantilevers based on several MOFs. It was concluded that a higher Poisson's ratio and Young's Modulus enhance the sensing response, whereas the density of MOFs negligibly affected the response.^[^ ^105^ ^]^ Li et al. compared the sensing performance of microcantilever sensors based on four different types of MOFs (i.e., MOF‐5, HKUST‐1, ZIF‐8, and MOF‐177) toward *p*‐xylene.^[^ ^106^ ^]^ The HKUST‐1 based microcantilever sensor exhibited a Langmuir‐type detection toward *p*‐xylene with a detection limit of around 400 ppb (Figure 8b,c), which is lower than that of the human olfactory threshold (470 ppb). The observed sensing behavior was associated with the appreciated host‐guest interaction that was justified by in situ diffuse reflectance infrared Fourier transform spectroscopy. Subsequently, similar investigations have been made on the fabrications of microcantilever sensors based on Cu‐BTC, Zn(BDC),^[^ ^107^ ^]^ ZIF‐8,^[^ ^108^ ^]^ ZIF‐7, ZIF‐65, ZIF‐71,^[^ ^109^ ^]^ MOF‐5,^[^ ^110^ ^]^ and even flexible MIL‐53^[^ ^111^ ^]^ for the sensitive detection toward a series of analytes including VOCs, aniline, CO, and CO_2_. It was envisioned that flexible MOFs exhibiting large lattice/volume changes would likely lead to unparalleled sensitivity toward some analytes compared to relatively rigid MOFs. However, it remains highly imperative to have a deeper fundamental understanding of the mechanical properties of MOFs and their correlation with framework dynamics upon the capture of exotic molecules. Moreover, special attention should also be paid to the gate‐opening behavior of flexible MOF thin films, other than only on MOF powders, whose crystalline structures undergo swelling under external stimuli such as light, temperature, pressure, and/or molecule adsorption.

#### 3.4.2 MOF‐Based QCM Gas Sensors

QCM sensor, composed of an AT‐cut quartz substrate with patterned circular electrodes on both sides, relies on the oscillator circuit that modifies the vibration frequency upon uptake of guest molecules by the top electrode. The shift of frequency can be approximately calculated using Sauerbrey's equation.^[^ ^104^ ^]^

(8) $$\Delta f\;=-\frac{2{f_{0}}^{2}}{A\sqrt{\rho \mu }}\Delta m$$

Where Δ*f* denotes the shift in frequency induced by the molecule adsorbed (Δ*m*); *f*_0_ represents the resonant frequency; *ρ* and *μ* represent the density and shear modulus, respectively. *A* is a constant based on the electrical and mechanical characteristics of the QCM. However, a higher frequency of operation (typically below 30 MHz) will increase the shift in frequency, while the limit of detection is governed by the signal‐to‐noise ratio. Early reports on the combination of MOFs and QCM studied the sorption kinetics or sensing behavior of various MOFs toward various vapors.^[^ ^36^, ^112^ ^]^ Kitagawa et al. described the integration of HKUST‐1 crystallites with varied sizes onto QCMs and studied their impact on the sorption kinetics. Their results indicated that the sorption kinetics toward diluted analytes depended largely on the crystal size, and a smaller size contributed to faster adsorption and thus a faster response irrespective of the gaseous analyte. Inversely, the significant sorbate‐sorbate interaction affected the sorption kinetics toward the analyte profoundly.^[^ ^113^ ^]^ Wöll et al. further clarified the relationship between the thickness of MOF film and the sorption and/or sensing behavior of HKUST‐1 based QCM sensors.^[^ ^114^ ^]^ They observed that the uptake of cyclohexane by the HKUST‐1 films was independent of the thickness of the MOF films (in the range of 52–690 nm). In contrast, the time for reaching the equilibrium increased quadratically with the film thickness. The mass transfer within the MOF film was governed by the intracrystalline diffusion rather than via the MOF surface barrier. The development of this field was also advanced by other groups.^[^ ^115^, ^116^, ^117^ ^]^ In a representative study, Fischer et al. successfully fabricated QCM sensors based on oriented Cu_2_(ndc)_2_(dabco) films obtained by step‐by‐step liquid‐phase epitaxy growth. The growth of Cu_2_(ndc)_2_(dabco) films with different preferential orientations was achieved by changing the terminated functional groups of the monolayers functionalized onto the Au‐coated QCM. The real‐time sensing curves confirmed an orientation‐dependent adsorption behavior of the textured Cu_2_(ndc)_2_(dabco) films in the [001] and [100] directions toward VOCs, including benzene, toluene, and *p*‐xylene. Specifically, the Cu_2_(ndc)_2_(dabco) film with a preferred [100] direction corresponding to a larger pore opening perpendicular to the underneath substrate favored a faster uptake of relevant molecules and thus contributed to a higher adsorption selectivity.^[^ ^118^ ^]^ In a separate study, Fischer et al. fabricated a series of QCM sensors based on different ZIFs (ZIF‐7, 8, 9, 67, 65, and 90) and systematically discussed the influences of pore size and geometry, surface functionality, and structural flexibility on their sensing performance.^[^ ^119^ ^]^ Chappanda, Eddaoudi, and co‐workers described the fabrication of HKUST‐1 based QCM sensors, which showed sensitive detection toward variable humidity concentrations (Figure 8d,e).^[^ ^117a^ ^]^ Sensitivity toward moisture can be substantially enhanced by 230% by compositing MOF with an optimal content of carbon nanotubes (CNTs). The CNTs/HKUST‐1 composite‐based QCM sensors outstood the previously reported humidity sensors. Following works conducted by several different groups concerned the fabrications of QCM gravimetric sensors based on ZIF‐8,^[^ ^120^ ^]^ MIL‐101,^[^ ^121^ ^]^ KAUST‐7/8,^[^ ^117b^ ^]^ and MOF‐14^[^ ^122^ ^]^ toward the detections of CO_2_/CH_4_, VOCs, SO_2_, and BTEX (benzene, toluene, ethylbenzene, xylene), respectively. The detection limit typically lies in the range of ppm‐level, with some exceptions within ppb‐level; the response generally takes several minutes. Compared with other devices, the QCM sensors feature great versatility that allows for facile operation under various conditions, as well as simplicity and commercial availability with cost‐effectiveness. This has made QCM devices valuable tools for probing in situ the sorption kinetics of MOF films toward a wide range of analytes, and even for studying the film growth mechanism of MOFs by layer‐by‐layer approach. However, the validity of the Sauerbrey equation is based on the assumption that MOF films possess identical acoustic properties similar to the quartz crystal.^[^ ^104^ ^]^ The reality is that such matching in acoustic property might deviate depending on the film thickness, the intrinsic mechanical property of MOFs, and the interfacial compatibility between the MOF and the underneath substrate. Thus, this point needs to be well clarified in future studies.

#### 3.4.3 MOF‐Based Microresonators for Gas Sensing

The working principle of gas sensing based on microresonators is conceptually analogous to that of the dynamic microcantilevers, except with an architecture comprising torsional cantilevers mounted on a paddle region. The microresonators are typically fabricated using clean‐room technologies involving multi‐step processing, including photolithography.^[^ ^35^, ^123^ ^]^ In an initial study conducted by Yaghi, Candler, and co‐workers,^[^ ^123^ ^]^ ZIF‐69 coated microresonator sensors were fabricated by drop‐casting the preformed MOF crystals onto the center paddle of the resonators. The frequency shift of the resultant resonators was substantially increased by around 78 times compared to that of the bare microresonator. It thus resulted in a higher sensitivity toward the targeted analyte. The detection limit of the ZIF‐69 coated resonator toward CO_2_ was derived to be around 0.13 pg, corresponding to a CO_2_ concentration of around 15 ppm. Younis, Eddaoudi, and co‐workers combined electrostatically actuated polymer microbeams with HKUST‐1 films obtained by inkjet printing, and demonstrated that such devices could act as electrical switches triggered by gas uptake.^[^ ^124^ ^]^ These sensors can switch from a “safe” indication to an “alarming” state once exceeding a pre‐set threshold mass. Measuring the differentiated amplitude gave the frequency shift and thus adsorbed mass, with a minimum detection mass of around 360 pg.

#### 3.4.4 MOFs‐Based Surface Acoustic Wave (SAW) Sensors for Gas Sensing

SAW sensors, first introduced by Wohltjen and Dessy in 1979,^[^ ^125^ ^]^ mainly consist of a couple of separated IDEs deposited on piezoelectric substrates. One IDE generates a SAW (also known as Rayleigh wave) and propagates along the surface. This wave exhibits a transverse motion and penetrates at least one wavelength depth into the underneath substrate. The second IDE picks up the acoustic wave and measures its variations in amplitude and phase. Compared with QCM sensors, SAW sensors typically operate under much higher frequencies (tens of MHz to GHz), which shows a much higher sensitivity. Coating MOFs within the propagation path of such SAW delay lines permits more accumulation in weight per area, resulting in higher sensitivity and even selectivity in some cases.^[^ ^126^ ^]^ Robinson et al. reported the fabrication of HKUST‐1 coated quartz SAW sensors utilized for the detection of humidity.^[^ ^127^ ^]^ The as‐fabricated sensors showed an ultrasensitive detection toward a wide range of water vapor (3–14 800 ppmv), outperforming commercially available humidity sensors. Time‐dependent response curves indicated that the response increased linearly at low moisture concentrations while increased abruptly along with the further condensation of water. Further evaluation toward the effect of film thickness on sensing performance suggested that the sensitivity first increased as the thickness increased. A maximal sensitivity was noticed with a MOF film thickness of around 200 nm. Thicker films added mass at a distance far from the surface that poorly coupled with the SAW but dampened the energy. Other studies including the coatings of MFU‐4, MFU‐4l,^[^ ^126^ ^]^ ZIF‐8,^[^ ^120^ ^]^ and ZIF‐67^[^ ^128^ ^]^ onto SAW sensors have also been reported for the detections of CO_2_, CH_4_, acetone, ethanol, and ammonia. For instance, Volkmer et al. grew MFU‐4 and MFU‐4l films with different pore sizes onto SAW sensors. They realized the fabrication of MOF‐based SAW sensors that exhibited very sensitive and fast (down to milliseconds) detection toward various gases (**Figure** **8**f,g).^[^ ^126^ ^]^ Specifically, the SAW sensor based on MFU‐4 film with an aperture size of 2.5 Å showed highly sensitive detections toward CO_2_, H_2_, NH_3_, H_2_O, and He, with a detection limit as low as 1 ppmv. Compared with other sensing technologies, the SAW sensors exhibit ultrafast detection, low cost, robustness, and high sensitivity toward tiny mass changes, holding great promise for studying the dynamics of gas uptake and diffusion in novel porous materials.

### 3.5 MOF‐Based Optical Gas Sensing

Interactions of light with solid‐state matters involve scattering, refraction, reflection, absorption, and potentially with emissions such as photoluminescence. Those interactions between light and MOFs with permanent porosity are typically guest molecule‐dependent.^[^ ^5^ ^]^ Photo‐responsive MOFs offer great opportunities for gas sensing through gas‐to‐optical and/or optical‐to‐electrical transduction routes in MOF‐based devices. Plenty of studies have been reported toward the design and synthesis of novel MOFs, emphasizing the host‐guest responsive photoluminescence via spectroscopic approaches.^[^ ^20^, ^39^, ^40^ ^]^ This field has recently been reviewed comprehensively.^[^ ^20^, ^39^, ^40^ ^]^ We herein focus only on the integration of MOFs onto devices targeted for gas sensing.

#### 3.5.1 MOF‐Based Fabry–Pérot Interferometers for Gas Sensing

**Figure 9:**
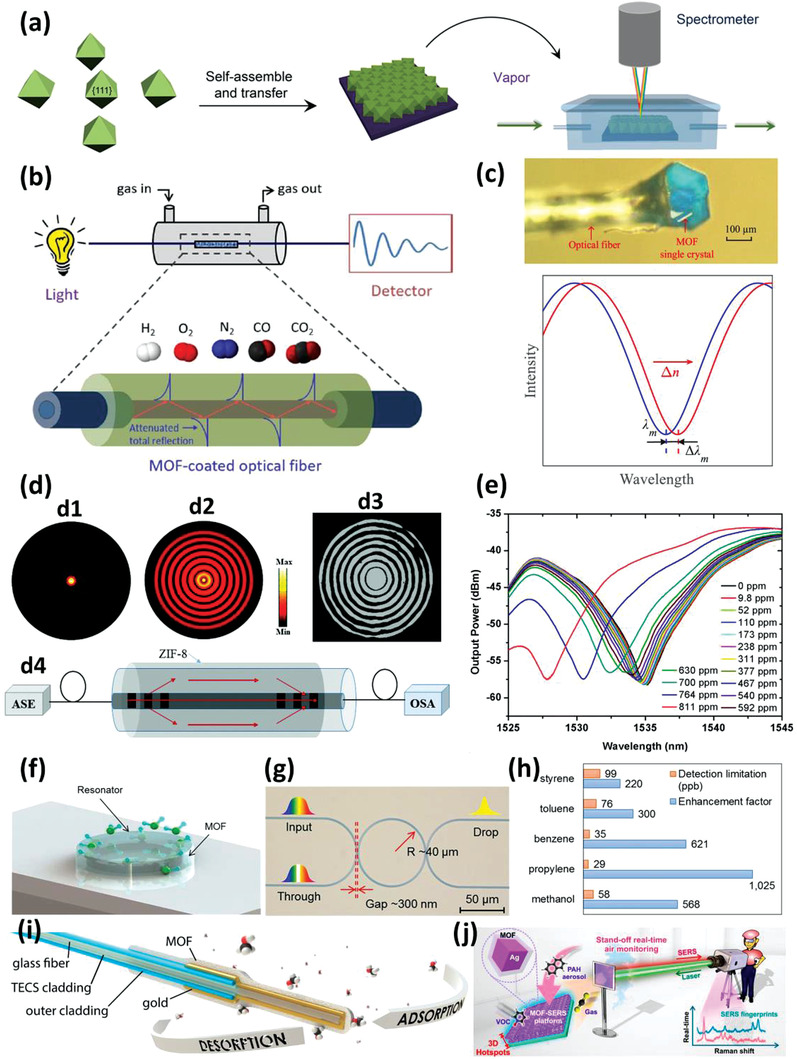
a) Schematic representation of self‐assembled UiO‐66 crystals‐based optical gas sensors. Reproduced with permission.^[^ ^133^ ^]^ Copyright 2019, American Chemical Society. b) Representative schematic diagram of MOF‐coated optical fiber sensors. Reproduced with permission.^[^ ^136^ ^]^ Copyright 2018, American Chemical Society. c) Optical paragraph of an optical fiber sensor based on HKUST‐1 single crystal. Below presents the illustration of wavelength shift due to the variation of RI. Reproduced with permission.^[^ ^138^ ^]^ Copyright 2019, American Chemical Society. d) Simulated optical power distribution of single‐mode fibers: d1) the core mode LP_01_; d2) the co‐propagating cladding mode LP_08_. Note that the LP01 mode can hardly detect the variation of RI of the coating on the cladding surface because of the confinement effect of light, whereas the evanescent field of LP08 manages to do so. d3) Near‐field image of the pattern of LP_08_ mode captured by an infrared camera. d4) Schematic representation of the proposed fiber sensor with LPFGs. e) Transmission spectra of the ZIF‐8 coated LPFG sensor toward variable concentrations of ethanol vapor. Reproduced with permission.^[^ ^139^ ^]^ Copyright 2020, Royal Society of Chemistry. f) Schematic representation of the MOF‐coated MRR. g) Schematic paragraph of the photonic MOF‐coated MRR with detailed components and connection. h) Detection limits and sensitivity enhancement factors at equilibrium of the ZIF‐8 coated MRR sensor toward different VOCs. Reproduced with permission.^[^ ^38^ ^]^ Copyright 2017, Springer Nature. i) Schematic representation of MOF‐coated SPR sensor. Reproduced with permission.^[^ ^145^ ^]^ Copyright 2017, American Chemical Society. j) Schematic display of stand‐off MOF‐SERS platform for air monitoring. Reproduced with permission.^[^ ^148^ ^]^ Copyright 2019, American Chemical Society.

The earliest MOF‐based devices for optical gas sensing rely on the refractive index (RI) variation of Fabry–Pérot interference.^[^ ^37^ ^]^ Inserting polarizable molecules into the cavity of MOF films displaces the vacuum and increases the overall RI, thus leading to a red‐shift of the interference peak. By monitoring the red‐shift of the interference of MOF films under exposure to volatile vapors by ultraviolet‐visible (UV–vis) spectrophotometer, for instance, one can estimate the concentration and/or type of relevant analyte. Férey et al. first demonstrated this concept using MIL‐101 thin films obtained by spin‐coating.^[^ ^44^ ^]^ The RI of the MIL‐101 film upon exposure to various molecules increased by around 30% derived from ellipsometry measurements. Hupp et al. demonstrated that ZIF‐8 films could also be utilized for guest‐sensitive Fabry–Pérot interferometers.^[^ ^37^ ^]^ ZIF‐8 thin films with variable thicknesses were grown in situ onto transparent glass substrates. The films with varying thicknesses showed different colors due to the diffraction and interference at the MOF‐air and MOF‐glass interfaces, corresponding to different optical wavelengths. An obvious red‐shift was noticed when exposing the ZIF‐8 film to different vapors, including propane, ethanol, and water. In separate studies, the same group reported the fabrications of HKUST‐1/silica composite films^[^ ^129^ ^]^ and ZIF‐8/Pt sandwich films,^[^ ^130^ ^]^ and demonstrated their usages for detecting selected analytes, including H_2_. In a follow‐up study, Lotsch et al. fabricated photonic sandwiched multilayers (also called Bragg stacks) composed of either ZIF‐8, HKUST‐1, or CAU‐1‐NH_2_ with TiO_2_.^[^ ^131^ ^]^ They demonstrated that the tandem MOF‐based Bragg stacks exhibited sensitive responses to various vapors. It was also indicated that cross‐sensitivity toward different analytes could be minimized via judicious choices of MOFs with varying polarities and principal component analysis of the color‐coded response. Flexible MOFs with breathing behavior have also been demonstrated for the detection of various vapors.^[^ ^132^ ^]^ Quite recently, Lu et al. reported the fabrication of optical MOF sensors based on self‐assembled UiO‐66 crystals (**Figure** **9**a) with tunable size and defects, and systematically investigated their sensing performance toward a series of gases including ethanol, methanol, acetone, cyclohexane, and hexane by a spectrometer (Figure 9a).^[^ ^133^ ^]^ The as‐fabricated sensors composed of well‐organized UiO‐66 crystals exhibited increased sensitivity by around 24.6% toward saturated ethanol vapor, compared to their counterpart. The crystal size showed a profound impact on the sensitivity, whereas it affected the response rate negligibly. Meanwhile, sensors based on crystals with missing ligands displayed a quicker recovery attributed to the fast sorption kinetics.

#### 3.5.2 MOF‐Based Waveguides or Optic Fibers for Gas Sensing

Waveguide‐based sensors, especially optic fiber ones, feature unique characteristics such as flexible design for in vivo/in situ analysis, online monitoring of various substances even in harsh environments, and insensitivity to external electromagnetic disturbances.^[^ ^134^ ^]^ The integration of porous MOFs on optic fibers leads to amplificated optical absorption with an increased RI correlated with the adsorption of gaseous analytes. Toda et al. developed HKUST‐1 coated optic fiber sensors with a light‐emitting diode as the light source and a photodiode as the detector.^[^ ^135^ ^]^ By monitoring the color variation of the MOF film in terms of depth and tone under exposure to variable humidity levels, they could reflect the concentration of water vapor. The sensor exhibited a nearly linear and fast response to trace moisture with a detection limit of 40 ppbv. The study demonstrated clearly how gas‐induced spectroscopic shift can be utilized in gas‐to‐optical transduction, with potential practical applications in gas monitoring. Kim et al. reported a ZIF‐8 coated optical fiber sensing platform, whose working principle relies on the variation of the refractive index under exposure to various analytes (Figure 9b).^[^ ^136^ ^]^ The as‐fabricated sensor exhibited a selective detection toward CO_2_ over N_2_, H_2_, CO, and O_2_, originating from the selective adsorption of ZIF‐8 toward CO_2_. The sensor also showed a fast response with tens of seconds at ambient conditions and high reversibility. Compared with the Fabry–Pérot sensing devices, such platforms based on optic fibers inherently showed an enhanced propagation of electromagnetic waves within the MOF layer, and therefore induced an apparent amplification of absorption and increased RI upon the capture of gases. In a follow‐up study, the same group introduced the post‐synthesis modification strategy to functionalize the Co‐doped ZIF‐8 coated optic fiber sensor with alkylamine, and investigated the sensing performance toward variable concentrations of CO_2_ under humid conditions.^[^ ^137^ ^]^ The alkylamine‐modified sensor based on optic fiber exhibited enhanced sensitivity and excellent stability toward CO_2_ under wet conditions. Distinctive from the above studies, Huang et al. coupled a HKUST‐1 single‐crystal with an optic fiber device (Figure 9c) and studied the uptake of nitrobenzene quantitatively.^[^ ^138^ ^]^ The dynamic response of the optic fiber sensor based on HKUST‐1 single‐crystal reflected a fast uptake of nitrobenzene within the MOF single crystal. Chiang et al. presented a manipulation of light to study the gas‐MOF interactions via employing optical fiber devices, as shown in Figure 9d.^[^ ^139^ ^]^ Long‐period fiber gratings (LPFG) were designed to excite a co‐propagating cladding mode LP_08_ at 1550 nm (Figure 9d, 2). Unlike the core mode LP_01_ (Figure 9d, 1), the evanescent field of LP_08_ on the cladding surface managed to “sense” the RI variation due to the gas‐MOF interaction. As such, the LPFG sensors integrated with ZIF‐8 film exhibited a higher sensitivity toward ethanol vapor in the range of 9.8–811 ppm (Figure 9e). This work provided a decent guideline for using MOFs in optic fiber and/or waveguide‐based sensing devices. Comparative studies on the usages of MOFs in LPFG or waveguide sensors have also been reported.^[^ ^140^ ^]^

#### 3.5.3 MOF‐Based Photonic Micro‐Ring Resonators for Gas Sensing

MRRs consist of a set of waveguides, among which at least one is a closed‐loop connected with light input and output (Figure 9f,g). Light of the resonant wavelength propagates in a circulating waveguide mode (CWM), originating from a total internal reflection of the curved boundary between mediums with different RIs.^[^ ^38^, ^141^ ^]^ Evanescent fields adjacent to the MRR are generated and can respond to changes of dielectric property and/or RI of the surrounding. The response of such sensing devices to variable gases is defined by the resonance wavelength shift caused by the variation of RI. A combination of porous MOFs with MRRs would greatly enhance such sensors’ sensitivity and even selectivity due to the preconcentrating and molecular sieving effects of MOFs. As exemplified by Zhao, Gu, and co‐workers, hydrophobic ZIF‐8 films have been integrated with photonic MRRs to preconcentrate the targeted analyte and increase their sensing immunity to humidity.^[^ ^38^ ^]^ The bare MRRs were fabricated using standard cleanroom technologies, which showed a nearly negligible shift in wavelength upon exposure to analytes. The fabrication of ZIF‐8 coated MRRs was realized by in situ film growth and subsequent chemical etching. The as‐fabricated compact MRRs were able to detect sub‐ppm toluene, styrene, propylene, benzene, and methanol, with detection limits ranging from 29 to 99 ppb (Figure 9h). Specifically, the ZIF‐8 coated MRR exhibited a sensitivity enhancement factor of more than 1000, compared with that of the bare MRR (Figure 9h). In the meanwhile, the sensors showed high immunity to humidity due to the hydrophobic nature of ZIF‐8. The presented results open novel perspectives for the fabrication of miniaturized MOF‐based photonic devices with high sensitivity.

#### 3.5.4 MOF‐Based Surface Plasmon Resonance (SPR) and Surface‐Enhanced Raman Scattering (SERS) Sensors for Gas Sensing

Surface plasmon resonance is essentially a charge‐density oscillation that typically occurs at the interface between two media with opposite permittivity, for instance, a metal and a dielectric (Figure 9i). Generally, the SPR sensor comprises an optical system that provides the light source, a transducing medium that interrelates relevant optical and chemical/biochemical domains, and an electronic system supporting the optoelectronic modules and allowing data processing.^[^ ^142^ ^]^ Variations in RI of the environment adjacent to a thin metallic layer (typically below 50 nm), such as Au, Ag, and Cu, lead to a shift in the resonance wavelength. The light from the optical system propagates through an optical fiber or waveguide until reaching the thin metallic layer coated fiber tip where surface plasmons can be generated. Such SPR probes based on optic fibers represent the highest miniaturization level compared with those based on the prism and/or grating couplers. Since its invention by Jorgenson and Yee,^[^ ^143^ ^]^ SPR sensors have been widely utilized in various sensing scenarios with good sensitivity and selectivity, including the identification and quantification of DNA and drug monitoring. In combination with MOFs, the SPR sensors have shown great promise for the detection of gaseous analytes. The first attempt conducted by Hupp and Duyne concerns the coating of HKUST‐1 on glass substrate patterned with Ag nanoparticle arrays, utilized to detect CO_2_ and SF_6_.^[^ ^144^ ^]^ A shift of the UV–vis extinction peak was monitored in real‐time once exposing the sample to the pure stream of the above two analytes. A red‐shift of the peak was observed due to the increase in RI of the sample upon gas uptake. After 37 circles’ growth, the MOF‐coated sensor showed a substantially enhanced signal (by 14‐fold) toward CO_2_, whereas it exhibited negligible enhancement toward SF_6_. Investigation on the dependence of peak shift on the MOF thickness indicated that the shift first witnessed a dramatic increase with the increase of the growth cycle. It then saturated after around 40 times’ growth of the MOF film. This observation was consistent with the fact that the plasmon‐induced electromagnetic field decays exponentially from Ag nanoparticle surface to the MOF layer. In a follow‐up study, Roeffaers et al. reported the fabrication of ZIF‐8 and ZIF‐93 coated SPR sensors based on optic fibers to detect alcohol vapors (Figure 9i).^[^ ^145^ ^]^ The bare SPR sensor exhibited no detectable signal toward the targeted analytes. However, the ZIF‐8 coated SPR sensor showed the lowest detection limit toward MeOH, that is, 2.5 ppm, whereas the ZIF‐93 coated SPR sensor exhibited the highest detection limit of 73 ppm toward *n*‐BuOH. Their sensing results also suggested that the ZIF‐93 coated SPR sensor recorded the highest sensitivity and selectivity toward MeOH, correlated with the higher affinity of ZIF‐93 toward MeOH at lower partial pressures. Dissimilarly, the ZIF‐8 coated SPR sensor showed a high sensitivity toward MeOH at high partial pressures, attributed to the molecular clustering effect of MeOH within the ZIF‐8 framework. Importantly, these SPR platforms can also serve as sensing systems to investigate the growth kinetics of MOF films and their sorption behaviors toward various gases.

An analogy methodology for gas sensing is the so‐called SERS based on their vibrational fingerprint of chemical bonds. Its working principle typically relies on the active metal sites that generate surface plasmons and electric fields. Incident photons allow for plasmon resonance excitation and cause the electric field to be enhanced both perpendicular and parallel to the surface.^[^ ^146^ ^]^ The Raman scattering is thus amplified, arising from the uptake of exotic molecules. An initial study conducted by Hupp and Duyne reported the fabrication of so‐called “films‐over‐nanospheres (FONs)” utilized to detect various aromatic molecules including toluene, benzene, nitrobenzene, or 2,6‐di‐*tert*‐butylpyridine through the SERS technique.^[^ ^147^ ^]^ The MOF‐coated samples after exposure to the above analyte were irradiated by two lasers and their spectra of the backscattered light were recorded accordingly. In such a fashion, the ZIF‐8 coated FON allowed recording the unique Raman fingerprint of each vapor and thus their identification. Oppositely, the bare FON failed to do so as it showed poor adsorptivity toward those vapors. Time‐resolved and concentration dependence measurements verified that those analytes were reversibly adsorbed within the surfaces of MOF crystallites exposed at grain boundaries. In a different study, Phang, Ling, and co‐workers described the preparation of Ag@MOF core‐shell nanostructures and their integration on SERS sensing platform for stand‐off and multiple detections of atmospheric airborne species (Figure 9j).^[^ ^148^ ^]^ The Ag@MOF core‐shell architectures were fabricated via self‐assembly and then deposited onto the SERS sensors, coupled with a stand‐off Raman system. The Ag@MOF coated SERS platform enabled rapid and quantitative detections toward ppb‐level gases and even aerosols 2–10 m away (Figure 9j). Impressively, the SERS sensing system with plasmonic architectures can record the unique fingerprints of multiple airborne aromatic hydrocarbons under an outdoor environment without obvious interference from the daylight. Quite recently, the direct usages of MOFs as active SERS substrates with molecule sieving effects have also been reported.^[^ ^149^, ^150^ ^]^ For instance, Zhao, Li, and co‐workers demonstrated that MOFs can serve as ideal SERS substrates to detect trace VOCs.^[^ ^150^ ^]^ The MIL‐100 SERS sensor showed a detection limit of 2.5 ppm toward toluene, outperforming the MIL‐88, MIL‐101, MIL‐125, and UiO‐66 SERS sensors. In combination with Ag nanoparticles, the MIL‐100 SERS sensor showed a detection limit of 0.48 ppb toward toluene, with an enhancement factor of 10^10^. Additionally, the MIL‐100 SERS sensor was able to detect gaseous analytes from the lung, showing great potentials for early in vivo diagnosis of lung cancer. These studies provided novel perspectives regarding the choices of SERS active substrates besides the noble metals and broadened the application of numerous MOFs in multitudinous SERS‐based sensing scenarios.

## 4 Conclusions and Perspectives

Progresses on the development of MOF‐based gas sensors have been significantly propelled in the past two decades by the advances in the design and synthesis of MOFs, film preparation approaches, as well as explorations of their physicochemical properties. Tremendous proof‐of‐concept demonstrations indicate that MOFs are promising candidate materials for advanced gas sensors, which hold great promises for monitoring indoor/outdoor air quality, industrial gas leakage, food freshness, and medical diagnostics. Judicious combinations of MOFs with unique features and various transduction architectures greatly benefit the sensing performance toward various gases in terms of sensitivity, selectivity, drifting, power consumption, etc. This review comprehensively summarizes the latest progress on the fabrication of MOF‐based gas sensors relying on different transduction mechanisms, including chemiresistive, capacitive/impedimetric, FETs/KPs, mass‐sensitive (microcantilever, QCM, microresonators, SAW), and optical methods (Fabry–Pérot interferometers, waveguides or optic fibers, MRR, and SPR/SERS). Meanwhile, the latest progress on the large‐area fabrication of MOF films that is essential for mass‐production of relevant devices yet absent from the literature is also reviewed. The roles of MOFs within those sensors and their impacts on sensing performance in terms of sensitivity, selectivity, and stability are discussed in detail. Although significant progress has been made toward the fabrication of MOF‐based gas sensors with salient performance and their usages in various sensing scenarios, this field remains rudimentary. More efforts are needed to address the challenges impeding their further mass‐production and commercialization.

1) The long‐term stability of MOFs under practical sensing environments might be problematic, especially under humid, acidic, or alkaline conditions with elevated pressure, temperature, etc. This issue will undoubtedly affect the sensing performance of MOF‐based sensors and eventually shorten their lifetime. Therefore, MOFs with highly robust coordination bonds connected by high valent metal centers (e.g., Ti^4+^ and Zr^4+^) and multidentate hydrophobic ligands would be good choices.
2) The poor fundamental understanding of MOF structures and structure‐property relationships restricts the further judicious design of MOFs with desirable physicochemical properties. It remains elusive how the host‐guest interactions are transduced into detectable signals. For instance, although dozens of intrinsically (semi)conductive MOFs have been synthesized by introducing planar and 2D *π*‐conjugated ligands with redox‐active sites, their charge transport mechanism within those 2D MOF architectures is not yet well‐understood, not to mention their stimuli‐responsive behaviors. Strategies to design and synthesize semiconductors with enhanced charge transport kinetics via doping of heteroatoms, strain/defect engineering, and compositing with other conductive materials might be accordingly referred. This will certainly enrich the fabrication of chemiresistive MOF‐based gas sensors with facile operation and cost‐effectiveness. Of course, intensive high‐throughput computational modeling coupled with the analytical evaluation of structure‐property correlations via in situ microscopic and spectroscopic techniques can be invaluable.
3) The integration of targeted MOFs in large‐area with good homogeneity, controllable thickness, orientation, appreciable adhesion/contacting, and precise positioning onto various miniaturized electronic devices, especially with complicated architectures (e.g., patterns and optical waveguides), still remains highly challenging. Development of generic approaches that are applicable to most MOFs and overcome the above issue would truly bring MOFs into the realm of microelectronics and/or optoelectronics targeted for diverse applications, including gas sensing.
4) Most of the studies mentioned above focus only on fabricating gas sensors based on a single MOF. Fabrication of sensor arrays integrated with multiple MOFs that show distinguishable selection toward different gases would be highly recommended for practical sensing scenarios that typically involve a complex gas mixture.
5) The sensing performance of MOF‐based sensors is yet to be improved by a combination of more advanced transduction architectures with multifunctional MOFs featuring appropriate porosity and dimensionality.
6) Relevant algorithms need to be developed or adopted for better data processing and pre‐training the MOF‐based sensors. As such, MOF‐based sensors with anomaly detection, self‐calibration, and lifetime prediction by machine learning can be feasibly afforded.

We believe that all these efforts will expedite the development of MOF‐based gas sensors with improved sensing performance, applicability, and touchable commercialization in the coming years.

## Conflict of Interest

The authors declare no conflict of interest.
